# Optimal circuit breaker allocation strategy in DC transmission-connected systems

**DOI:** 10.1038/s41598-024-74166-1

**Published:** 2024-10-17

**Authors:** Beopsoo Kim, Insu Kim

**Affiliations:** Department of Electrical and Computer Engineering, Inha University, Incheon, Republic of Korea

**Keywords:** HVDC, Circuit breaker, Fault, Energy grids and networks, Power distribution

## Abstract

The purpose of this study is to provide data for the best possible circuit breaker allocation in power grids with HVDC transmission while taking circuit breaker failure into account. The lack of a suitable selection of HVDC circuit breakers is one of the primary obstacles to establishing an appropriate protection system for HVDC transmission. This research focuses on finding the optimal circuit breaker allocation strategy with respect to the transient response of the system, especially when the grid operator is using existing circuit breakers, rather than on the necessary steps to improve the technology of circuit breakers. Case studies are simulated using PSCAD/EMTDC in order to verify the transient response of the transmission system corresponding to the allocation of circuit breakers and the circumstance of circuit breaker failure. It is crucial to remember that there isn’t a strategy that is always the best. Consequently, a detailed analysis of the suggested approach is required, taking into account the benefits and drawbacks from the standpoints of fault clearing, power system planning, and operational factors. In the context of HVDC transmission, the authors hope that their research will spark and start conversations about circuit breaker allocation strategies.

## Introduction

### Motivation

As society develops, the demand for electrical energy increases, leading to the need for effective bulk power transmission. In order to meet the requirements of power transmission, the high-voltage direct current (HVDC) transmission method has been proposed by academia and industry as the key transmission method. Although the demand for the DC-based transmission method has led to the power grid as AC-DC mixed, the development of a circuit breaker (CB) considering the change of power grids - considering the AC side and DC side - is still inadequate. As the precedent study^1] and [2^, showed that the CB allocated on the AC side can properly protect the HVDC transmission and receiving equipment. However, the method proposed^1,2^ requires the whole area to be disconnected from the transmission side afterwards, this strategy is not an inappropriate way to protect and coordinate on mixed AC-DC transmission and distribution system. For fast detection and protection of DC fault current occurring on DC transmission system, the application of DC-based CB is the most appropriate way^3^, proposed an optimized fault detection and location framework for underground cables based on a discrete wavelet transform, which incorporates area detection, faulty section identification, and fault location, utilizing relay coordination optimization through Newton–Raphson analysis and modeling of DWT traveling times to achieve rapid and accurate fault detection and location in 0.01 s for 132 kV/132 kV/11 kV and 16-node underground cable networks. AC-based CBs have been used the way to manage DC fault current considering the contemporary technological advancement and economic factor^4^. However, the cut-off method using AC-based CB, the limitations mentioned in^1,2^ are clear. Due to the limitations, various CBs are designed for HVDC transmission systems, and IEEE also proposes standards^5,6^. Furthermore, in the future power system, issues have arisen regarding grid connection delays that may occur due to the complexity and decentralization of the power system as a result of the implementation of renewable energy based distributed generators (DGs)^7,8^. Similarly, the effects on the system in the event of a failure in the HVDC and multi-terminal direct current (MTDC) interconnected systems have also been presented^9^. showed that the allocation of power semiconductor-based breaker or hybrid breaker with reactors is the appropriate for the bipolar HVDC transmission on ground return and back-to-back link. And^10^ proposed that the transient phenomenon seen in accidents on the DC and AC sides was analyzed, and it was revealed that attention should be paid to the protection and cooperation depending on the characteristics of the transformer grounding and neutral point grounding resistance. In addition, IEEE PES presented problems that may occur in DC transmission as one of the tasks for protection in microgrids through^11^.

### Previous studies

#### Development of alternative solutions of CBs

Recently, due to the excessively high cost of circuit breakers designed for DC transmission and distribution systems, some researches for alternative solutions are conducted^12,13^. There are generally two methodologies and current research trends as outlined as follows:

##### Solid-State Circuit Breakers (SSCBs)

The recent research trend in solid-state circuit breakers (SSCBs) highlights their growing importance in modern electrical systems, particularly in low-voltage direct current (LVDC) and medium-voltage direct current (MVDC) applications. SSCBs offer advantages such as fast interruption, arc-less operation, and enhanced reliability compared to traditional mechanical circuit breakers. This trend is driven by advancements in power electronics and the increasing demand for efficient and reliable power distribution systems. Design innovations in SSCBs focus on optimizing power device performance and topology, with recent designs leveraging semiconductor power devices like insulated gate bipolar transistors (IGBTs) and silicon carbide (SiC) MOSFETs. A novel SSCB design employs transient current commutation to eliminate parasitic inductances and voltage oscillations, enhancing reliability and extending device lifetime^14^. The integration of snubber branches, such as the MOV-C snubber, helps manage voltage rise rates and suppress high-frequency oscillations during the breaking process, particularly effective in low-voltage DC systems^15^. SSCBs find application in various areas, including motor control centers (MCCs), aviation and hybrid electric propulsion systems, and military and shipboard applications. In MCCs, SSCBs integrate multiple functionalities, such as soft start and overload protection, into a single device, reducing system bulk and enhancing operational intelligence^16^. For aviation, SSCBs are designed for high-density and high-efficiency applications, where they can limit peak fault currents without additional inductors, thus improving power density and system reliability^17^. The United States Navy utilizes SSCBs in shipboard DC power distribution systems to meet the demands for increased power and efficiency, benefiting from SSCBs’ fast operation and high power handling capabilities^18^. Despite their advantages, SSCBs face several challenges. Managing transient recovery voltage (TRV) is crucial for the reliable operation of fast circuit breakers. The integration of superconducting reactors has been shown to significantly reduce TRV, offering a promising solution for modern power grids^19^. Extending the lifetime and reliability of SSCBs is another key focus, with strategies such as optimized turn-off solutions and the use of robust snubber designs being explored to enhance reliability without compromising performance^15,16^. As SSCBs continue to evolve, they are expected to play a pivotal role in the future of power distribution systems. Modular SSCB designs enhance scalability and fault tolerance, making them suitable for both LVDC and MVDC grid applications. These designs often incorporate bidirectional capabilities, allowing for more flexible and efficient power management^20^.

##### Hybrid DC Circuit Breakers

Hybrid DC Circuit Breakers are pivotal in modern DC power systems, offering rapid fault isolation and current limiting capabilities essential for maintaining system stability and safety. Recent research has focused on enhancing the performance, efficiency, and reliability of these breakers through innovative designs and technologies. A novel hybrid DC circuit breaker with current limiting capabilities has been proposed to address the rapid rise of fault currents in MMC-HVDC systems. This design incorporates a metal-oxide arrester and an energy dissipation circuit to reduce fault isolation time and thermal effects, enhancing reliability and reducing capacity requirements^21^. Another innovative topology involves a bi-directional current-limiting hybrid DCCB, which uses power electronic switches to transition between low and high reactance modes, effectively limiting current during mechanical switch opening^22^. Additionally, a bipolar hybrid DC circuit breaker with surge arresting mechanisms has been developed to manage voltage rises during current disruptions, minimizing arcing and mechanical switch erosion^23^. The recovery behavior of mechanical switching paths in hybrid circuit breakers has been studied, revealing that the recovery speed depends on the conductance of the switching path, which can be optimized by adjusting parameters such as load current and contact distance^24^.

Performance optimization and addressing challenges have been key areas of development. The influence of stray inductance on the breaking performance of DC circuit breakers has been studied, providing insights into designing high-capacity parallel interruption DC circuit breakers for flexible DC transmission systems^25^. A novel hybrid DC circuit breaker design eliminates solid-state switches in the main current path by using a reverse-biased voltage source, reducing thermal management challenges and facilitating fast current commutation^26^.

#### The current state of DC protection standardization

The current state of DC protection standardization is characterized by ongoing efforts to harmonize and develop standards that address the unique challenges posed by DC systems. IEC plays a pivotal role in this process, with the IEC 60,255 series being developed to address the requirements and testing of protection devices with digital inputs and outputs, ensuring compatibility with existing standards like IEC 61,850 and IEC 61,869^27^. However, national standards, such as the Korean Standards (KS), often adopt IEC standards but lack integration with domestic group standards like SPS, highlighting the need for a more cohesive approach to standardization^28^. DC microgrids present unique protection challenges due to the absence of natural zero-crossing in DC currents, which complicates fault detection and isolation. This necessitates the development of specialized protection devices and schemes^29^. The metrology requirements for DC protection schemes are critical for their practical implementation, yet they are often overlooked in existing standards, creating a gap that needs to be addressed to ensure accurate fault detection and system reliability^30^. To meet these challenges, the development of solid-state circuit breakers and other advanced protective devices is crucial for effective DC microgrid protection. These technologies offer faster response times and improved reliability compared to traditional electro-mechanical breakers^31^. Practical approaches to meeting standard requirements while maintaining cost-effectiveness are being explored. The use of Commercial-Off-The-Shelf (COTS) components in designing protection systems for DC Zonal Electrical Distribution Systems (ZEDS) demonstrates one such approach^32^.

### Contributions and applications

Regrettably, it is rather unsatisfactory that the topic of circuit breaker placement strategies was not covered in the related research, and it was not convincing in terms of specifics, as it only provided tasks and instructions. Therefore, due to the limitations of power semiconductor technology, this paper has conducted a study that takes into account the fact that the transient state is different depending on whether the fault location is on the DC or AC side in the HVDC transmission system, which currently responds to system failure situations with AC circuit breakers. At present, the optimal placement of circuit breakers in the event of a single line-to-ground (SLG) fault in a transmission line, which is a representative accident that frequently occurs in the system, is being considered. In addition, through this study, we hope to develop a circuit breaker placement strategy for economical and safe system protection.

The subsequent sections of this work are structured as follows. The problem statement of this work is presented in Sect. 2. The modeling process with PSCAD/EMTDC is described in Sect. 3. Section 4 presents the findings of case studies, which analyze the failure of CB as well. In Sect. 5, the findings are proposed.

## Problem statement

**Fig. 1:**
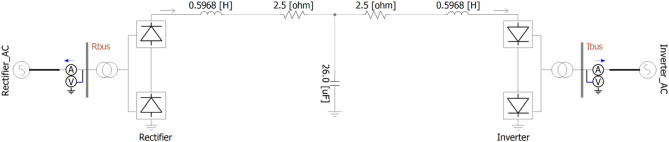
The monopolar HVDC transmission system for case studies^33,34^.

**Fig. 2:**
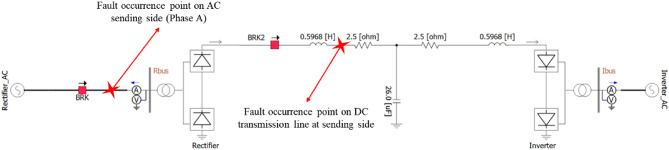
The fault occurrence point and CB locations of this study.

In this paper, based on the basic monopole HVDC transmission system defined by Cigre through^33] and [34^, the authors assume and simulate SLG faults in transmission lines that typically occur in transmission lines on the DC and AC sides of the transmission end. Through case studies, the transient state response of the system is checked for each circuit breaker allocation strategy and the allocation strategy is evaluated. For this case study, based on^33] and [34^, faults are simulated using the test system shown in Fig. 1 modeled by PSCAD/EMTDC.In addition, it would be beneficial to establish the CB allocation strategy and simulate the transient response that will occur when such a fault occurs. To provide comprehensive information on the simulation of the research in this paper, we proposed Fig. 2, and Table 1. Figure 2 provides the precise location of faults and the positions of CBs. Table 1 provides a summary of the fault occurrence point in this study and the corresponding CB locations.

**Table 1:** The type of faults that occurred in suggested transmission system and CB allocation strategies.

Fault type	CB allocation strategy
SLG fault on AC transmission line at sending side	CBs are installed on both the AC and DC sides
	CB is only on the AC side
	CB is only on the DC side
SLG fault on DC transmission line at sending side	CBs are installed on both the AC and DC sides
	CB is only on the AC side
	CB is only on the DC side

## Transmission system modeling using PSCAD/EMTDC

### Transmission side rectifier modeling

**Fig. 3:**
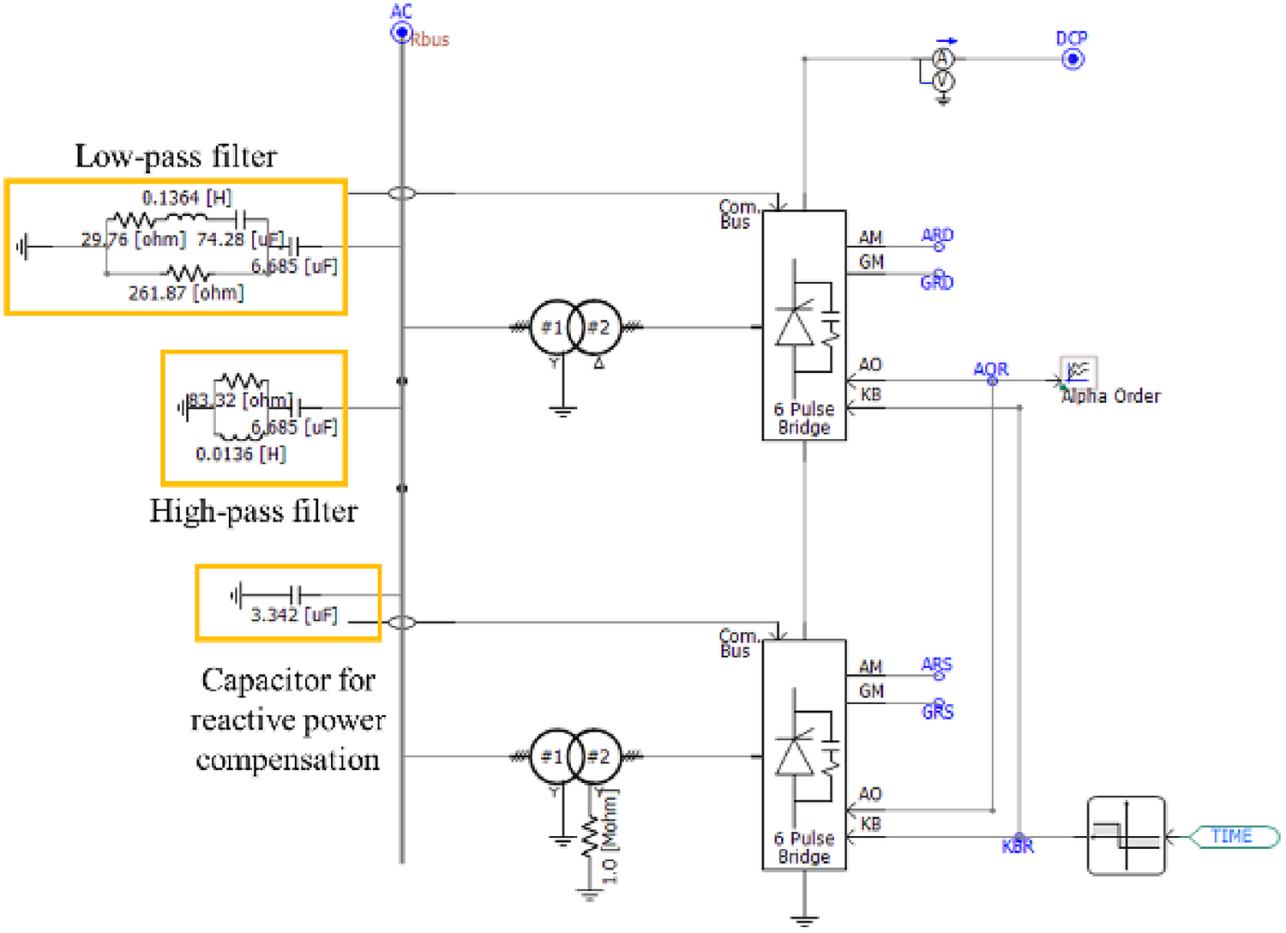
The modeling of rectifier.

The transmission-side rectifier section can be modeled using PSCAD/EMTDC as shown in Fig. 3; in this rectifier model, the twelve-pulse bridge is made up of two six-pulse bridges. At this point, the step-down transformer on the top side converts and transmits power to the secondary side through Wye-Delta connection, and the step-down transformer on the bottom side converts and transmits power to the secondary side through Wye-Wye connection. This plays the role of creating a phase difference for the implementation of a twelve-pulse bridge, and is finally completed through the operation of the six-pulse bridge on the top side and the six-pulse bridge at the bottom-side with a time delay. In addition, a low-pass filter and a high-pass filter are applied to remove the 11th and 13th harmonics that occur during a fault, and a capacitor is added to compensate for reactive power. And the detailed information of the rectifier is presented in Table 2.

### Receiving side inverter modeling

**Fig. 4:**
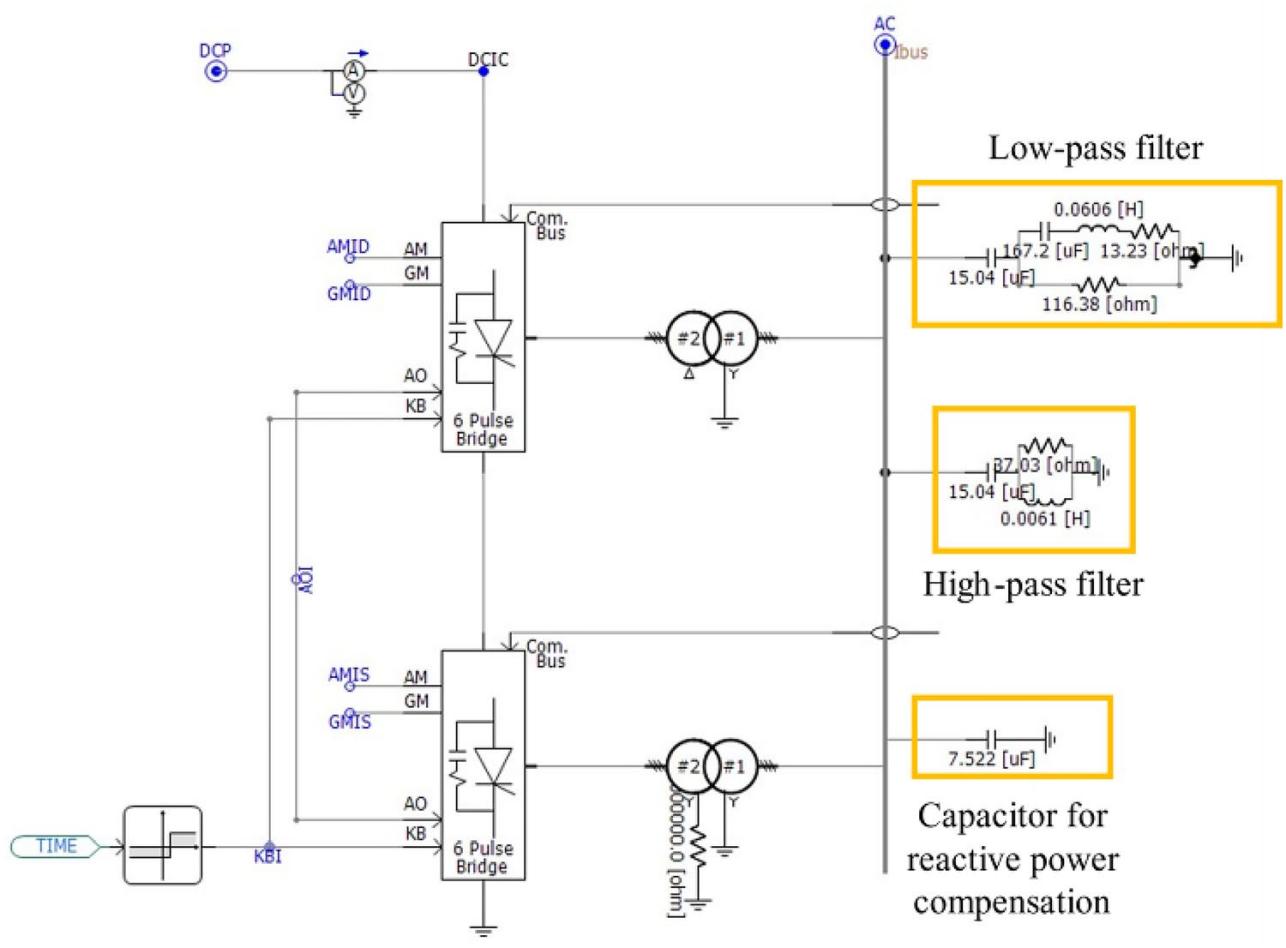
The modeling of inverter.

The receiving-side inverter is modeled as the same structure as the rectifier part of the transmitting side presented in the Sect. 3.1. In the same procedure as the rectifier, the process of converting DC voltage and current into AC voltage and current is operated in the form of the twelve-pulse bridge by two six-pulse bridges; the upper-side six-pulse bridge adopts Delta-Wye connection, and the lower-side six-pulse bridge adopts Wye-Wye connection to generate the phase difference. The conversion back to three-phase AC power is performed based on the operating time difference between the phase difference and the 6-pulse bridge. High-pass and low-pass filters and power factor correction capacitors are used to remove the harmonics generated in the process and to compensate for reactive power. The structure of the inverter is shown in Fig. 4, and detailed specifications are presented in Table 2.

**Table 2:** The detailed data of rectifier and inverter.

	Rectifier	Inverter
AC base voltage	345 kV	230 kV
Base MVA	100 MVA	100 MVA
Voltage source (p.u)	$1.088\angle 22.18^\circ$	$0.935\angle - 23.14^\circ$
Nominal DC voltage	500 KV	500 kV
Nominal DC current	2 kA	2 kA
Source impedance	*R* = 3.737 Ω L = 0 H	*R* = 0.7406 Ω L = 0.0365 H
System frequency	50 Hz	50 Hz
Minimum angle	$\alpha =15^\circ$	$\gamma =15^\circ$

## Case study and results

In this case study, as described above, the transient situation of the power system that occurs according to the CB placement strategy when the ground fault occurs in a transmission line on the AC and DC sides was simulated by PSCAD/EMTDC. In the case of a ground fault in the transmission line on the AC side, a ground fault in phase A was simulated. A fault occurred one second after the start of transmission. The CB was opened, the fault was removed 0.05 s later, and the transmission system was reconnected at 1.1 s.

### CBs are installed on both the AC and DC sides

#### DC transmission line-to-ground fault

##### Circuit breaker operated well on fault duration

**Fig. 5:**
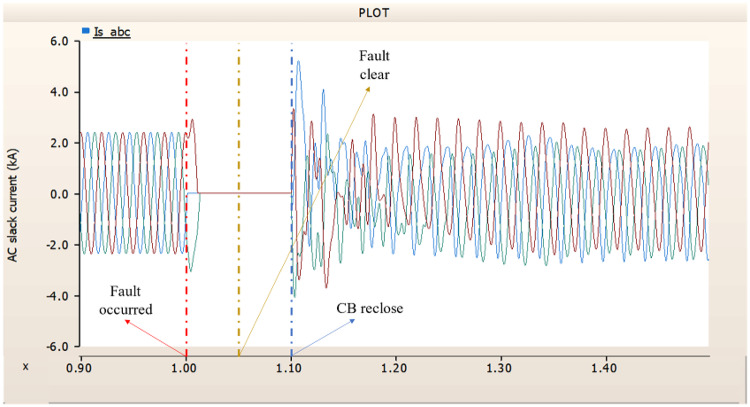
The output pattern of sending side end generator on DC transmission line-to-ground fault with CB allocated AC and DC side both.

**Fig. 6:**
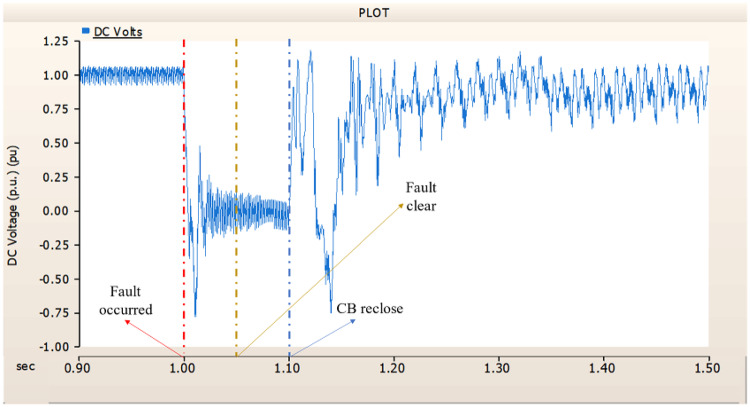
The DC voltage output pattern of rectifier on DC transmission line-to-ground fault with CB allocated AC and DC side both.

**Fig. 7:**
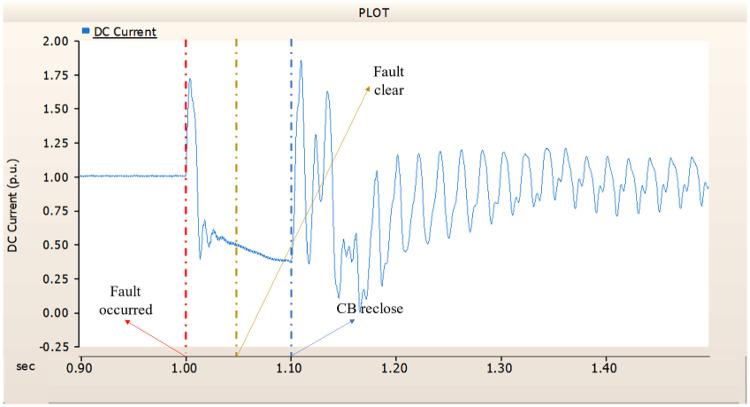
The DC current output pattern of rectifier on DC transmission line-to-ground fault with CB allocated AC and DC side both.

When a ground fault occurs in a DC transmission line and circuit breakers are installed on both the DC and AC sides, the transmission side generator appears to recover its original output, but the rectifier shows a very slow recovery. Both the DC voltage and current of the rectifier return to around 1pu, but the width of the output voltage and current is not constant, showing a phenomenon of poor stability. This seems to be due to the characteristics of the controller presented in [33] and [34]. The implemented controller uses a phase-locked oscillator based on the $\alpha /\gamma$ ratio, the three-phase voltage of the power generation source, and the information exchange between the rectifier and the inverter. The error is controlled using the proportional integrator control technique. However, at this time, due to the circuit breakers on both the DC and AC sides, the allowable error range in the controller is exceeded and recovery is slow, resulting in poor quality. The transient response of the generator at the transmission end is shown in Fig. 5, and the transient response of the rectifier is shown in Figs. 6 and 7.

##### Circuit breaker failed to fault clearance

The three cases that comprise this chapter’s breakdown of CB failures are also applied to 4.1.2.2, and are compiled into Table 3. Initially, both the AC and DC side CBs that were fitted failed to operate. Second, the CB that was installed on the AC side worked properly, but the CB that was installed on the DC side did not. Third, the CB that was mounted on the AC side did not function, while the CB that was mounted on the DC side did. In this simulation, CB operates at 1.00 s. And the failure of CB occurred 0.02 s (i.e., 3 cycles) later.

**Table 3:** The availability of circuit breakers failure (O : operated well / X : failed to fault clearance).

	Case 1	Case 2	Case 3
**AC Side**	X	O	X
**DC Side**	X	X	O

**Fig. 8:**
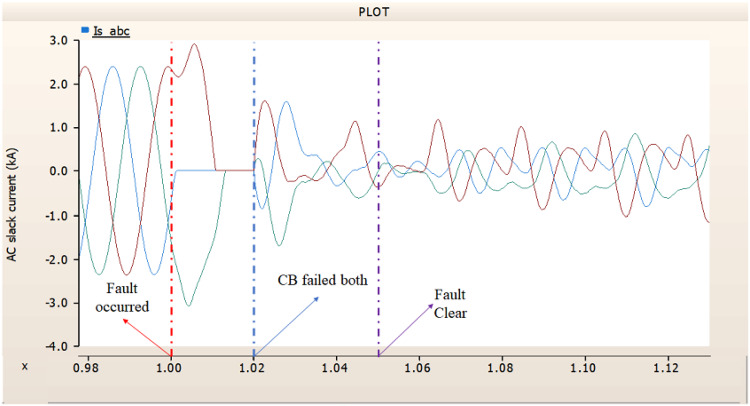
The output pattern of sending side end generator on DC transmission line-to-ground fault with CB allocated AC and DC side both. During the fault duration, both the AC side and DC side CB failed.

**Fig. 9:**
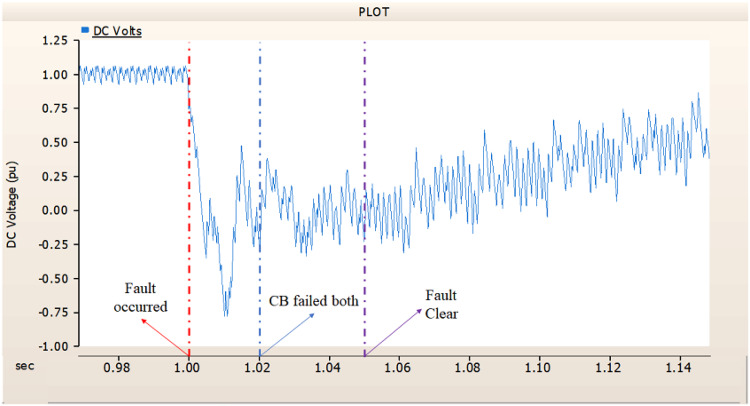
The DC voltage output pattern of rectifier on DC transmission line-to-ground fault with CB allocated AC and DC side both. During the fault duration, both the AC side and DC side CB failed.

**Fig. 10:**
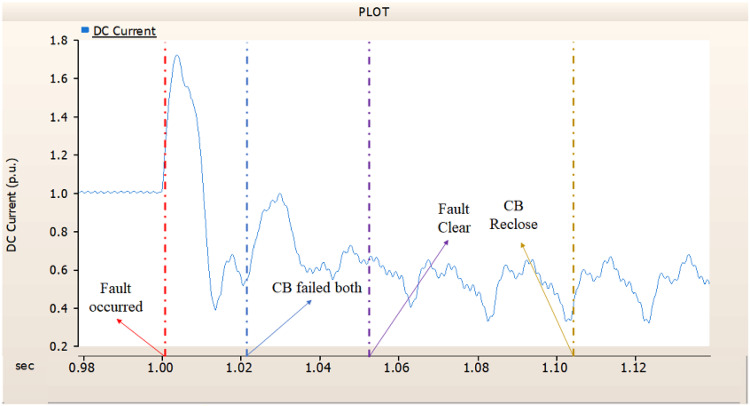
The DC current output pattern of rectifier on DC transmission line-to-ground fault with CB allocated AC and DC side both. During the fault duration, both the AC side and DC side CB failed.

In the first case, unlike 4.1.1.1, Fig. 8 shows the current output pattern of sending side generator is distorted on all phases. Also, Figs. 6 and 7 showed the tendency to return to the original output pattern, Figs. 9 and 10 did not show the tendency to return to the original output pattern. Considering the failure of CBs, the CBs’ failure made controllers can not control the error made by fault.

**Fig. 11:**
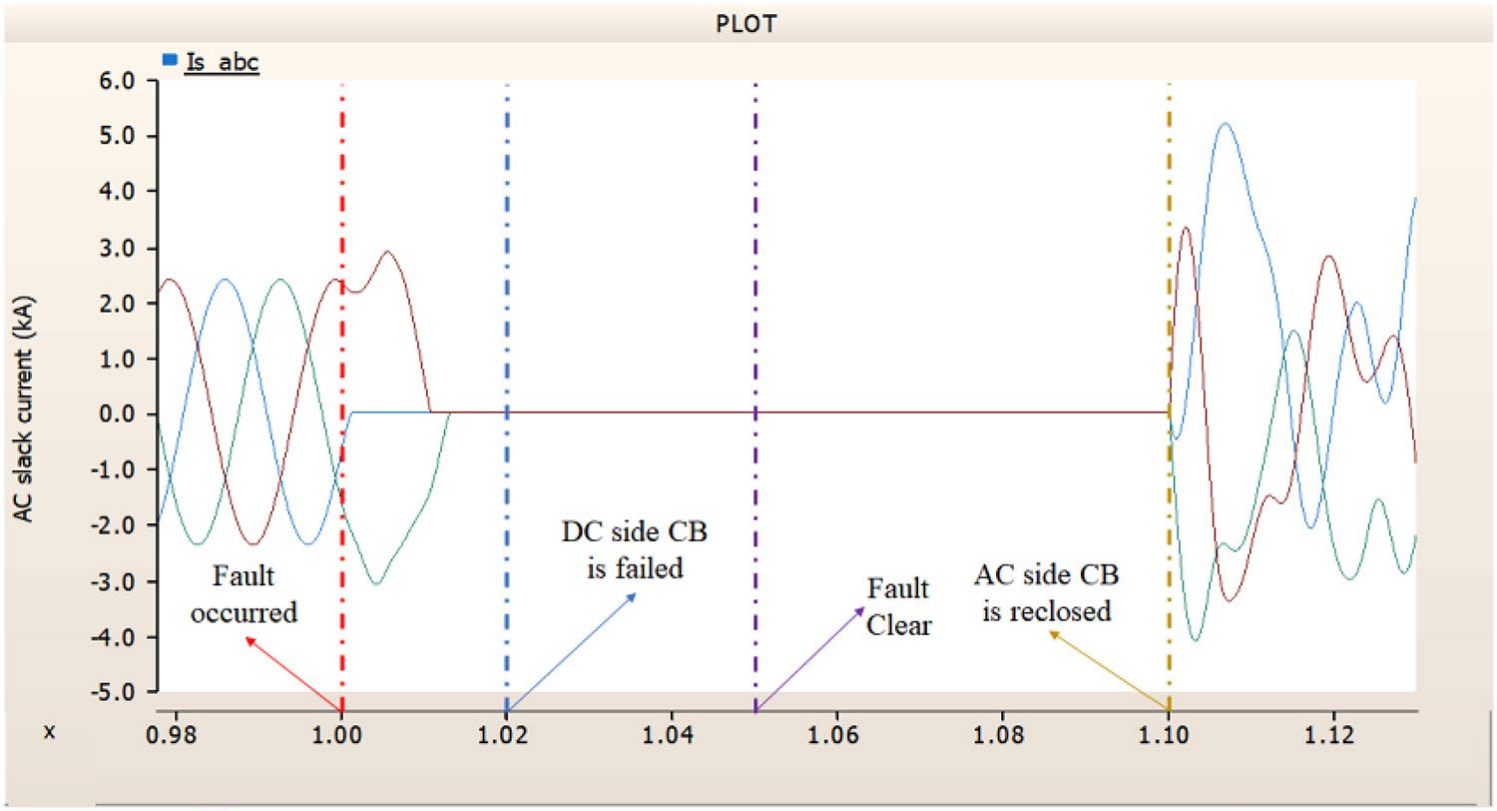
The output pattern of sending side end generator on DC transmission line-to-ground fault with CB allocated AC and DC side both. The AC side CB operated well, but the DC side CB failed on fault duration.

**Fig. 12:**
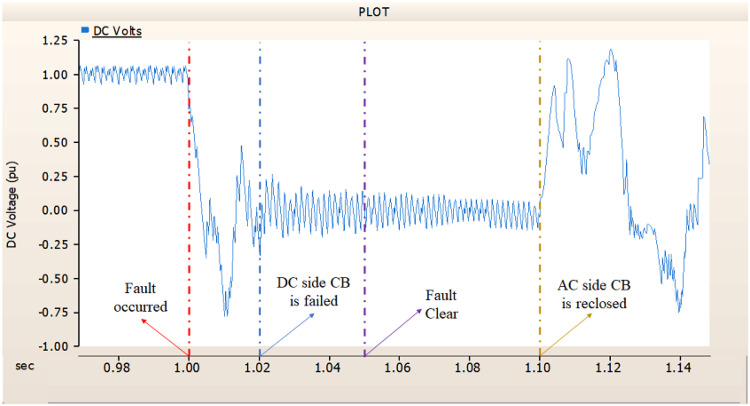
The DC voltage output pattern of rectifier on DC transmission line-to-ground fault with CB allocated AC and DC side both. The AC side CB operated well, but the DC side CB failed on fault duration.

**Fig. 13:**
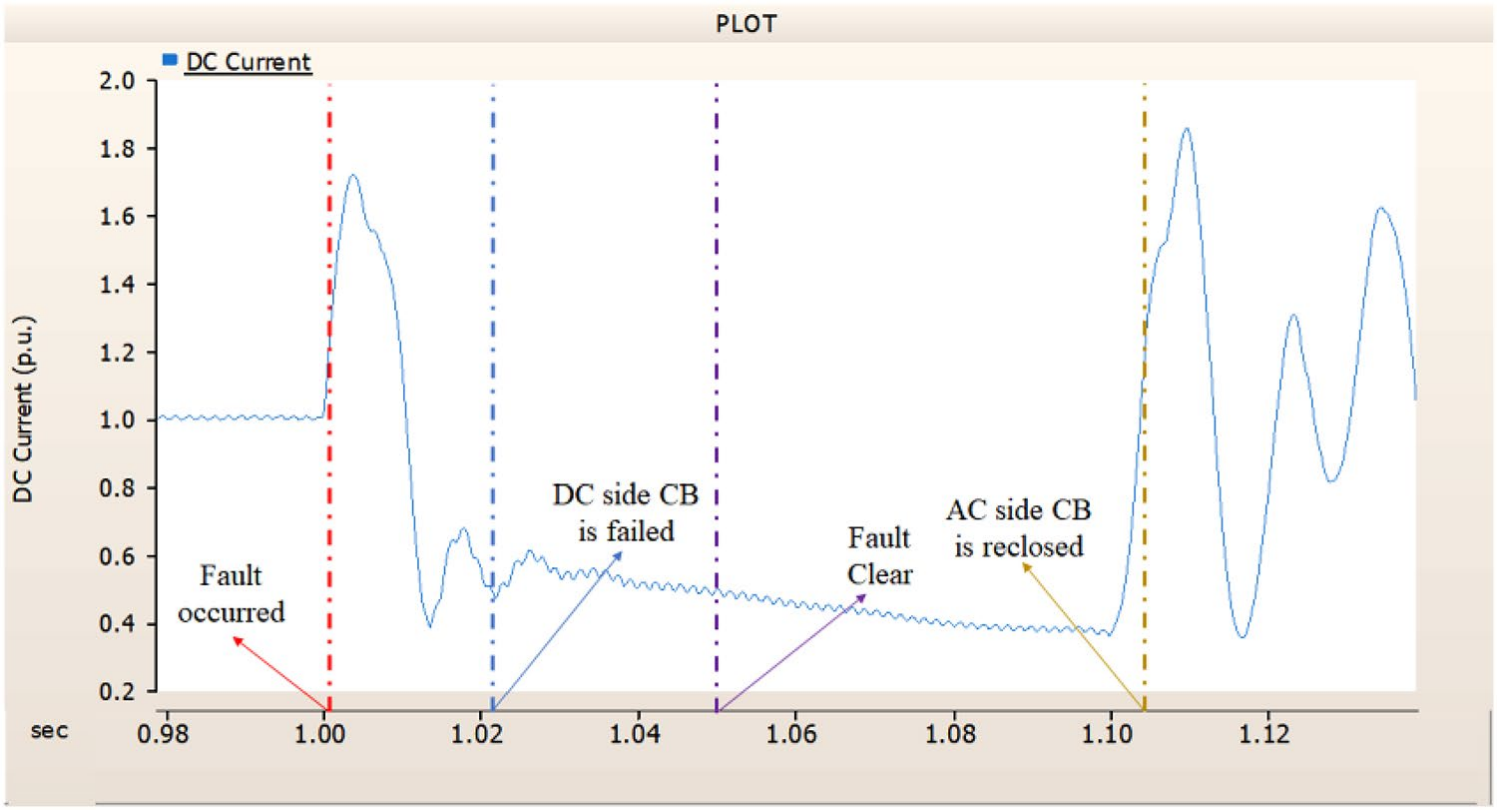
The DC current output pattern of rectifier on DC transmission line-to-ground fault with CB allocated AC and DC side both. The AC side CB operated well, but the DC side CB failed on fault duration.

In the second case, in Fig. 11, the output pattern of the sending side synchronous generator showed distorted current outputs although the AC side CB operated well. Also, considering the output pattern of rectifier’s DC voltage and DC current output pattern illustrated on Figs. 12 and 13, the output can not recover the prefault output patterns. However, the outputs shows arising from after the reclose of AC side CB, the proper control strategy of synchronous generator and rectifier both.

**Fig. 14:**
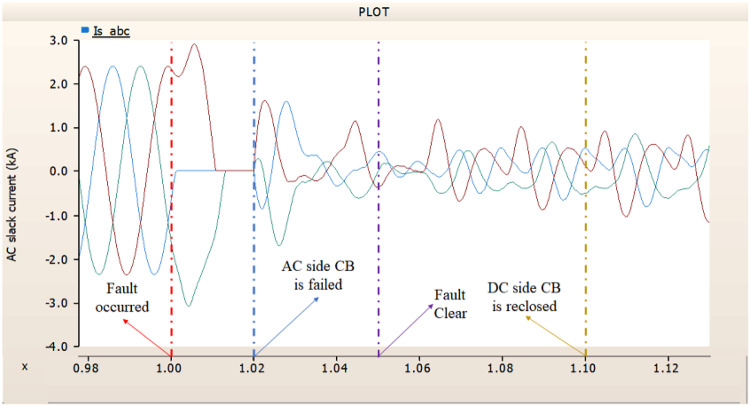
The output pattern of sending side end generator on DC transmission line-to-ground fault with CB allocated AC and DC side both. The DC side CB operated well, but the AC side CB failed on fault duration.

**Fig. 15:**
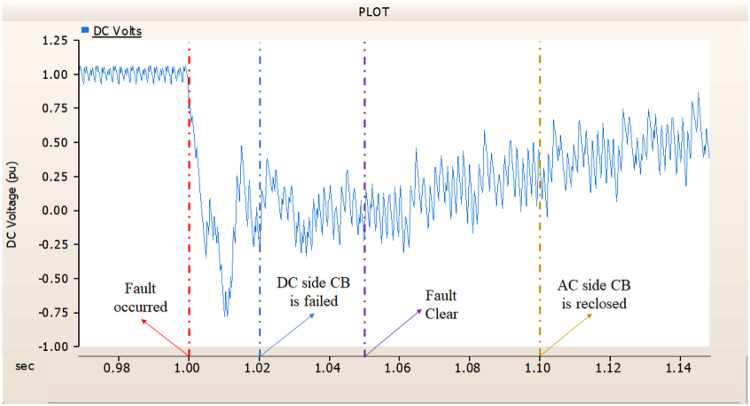
The DC voltage output pattern of rectifier on DC transmission line-to-ground fault with CB allocated AC and DC side both. The DC side CB operated well, but the AC side CB failed on fault duration.

**Fig. 16:**
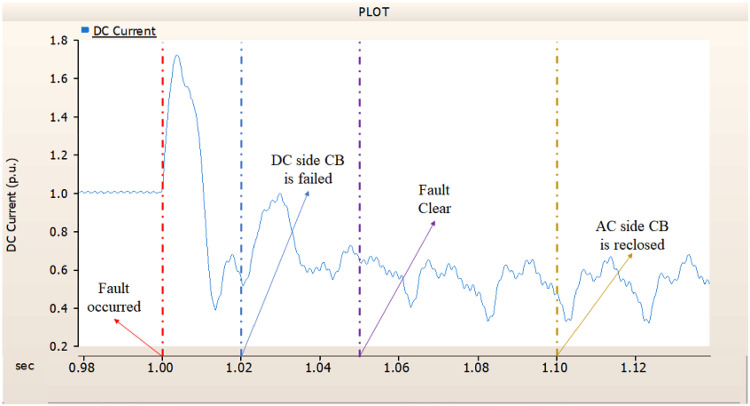
The DC current output pattern of rectifier on DC transmission line-to-ground fault with CB allocated AC and DC side both. The DC side CB operated well, but the AC side CB failed on fault duration.

In the third case, in Fig. 14, the output pattern of the sending side synchronous generator showed distorted current outputs. However, compared with the output pattern of the second case (i.e., Fig. 11), the peak value of current is smaller than the second case although the AC side CB failed. Also, considering the output pattern of Figs. 15 and 16, the DC voltages recovering the output pattern for returning prefault pattern, the DC currents can not recover the output pattern. Considering these results, the collaboration of rectifier and synchronous generator is essential on after fault control.

#### AC transmission line SLG fault on phase A

##### Circuit breaker operated well on fault duration

**Fig. 17:**
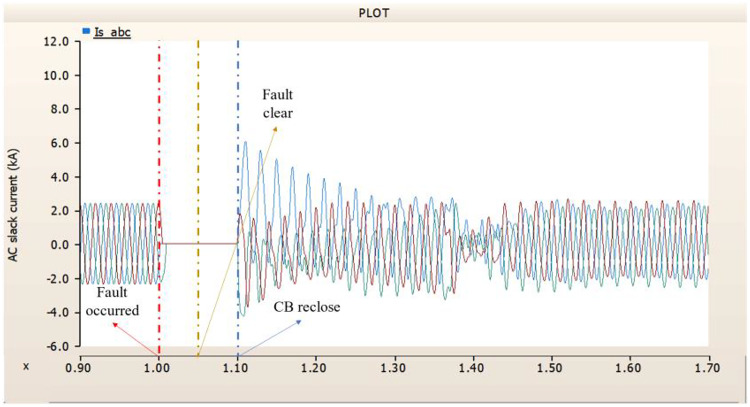
The output pattern of the sending end generator on AC transmission line to ground fault on phase A with CB allocated AC and DC side both.

**Fig. 18:**
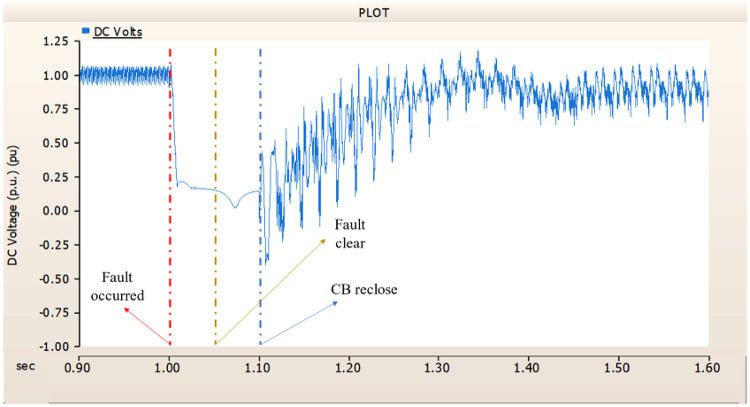
The DC voltage output pattern of rectifier AC transmission line to ground fault on phase A with CB allocated AC and DC side both.

**Fig. 19:**
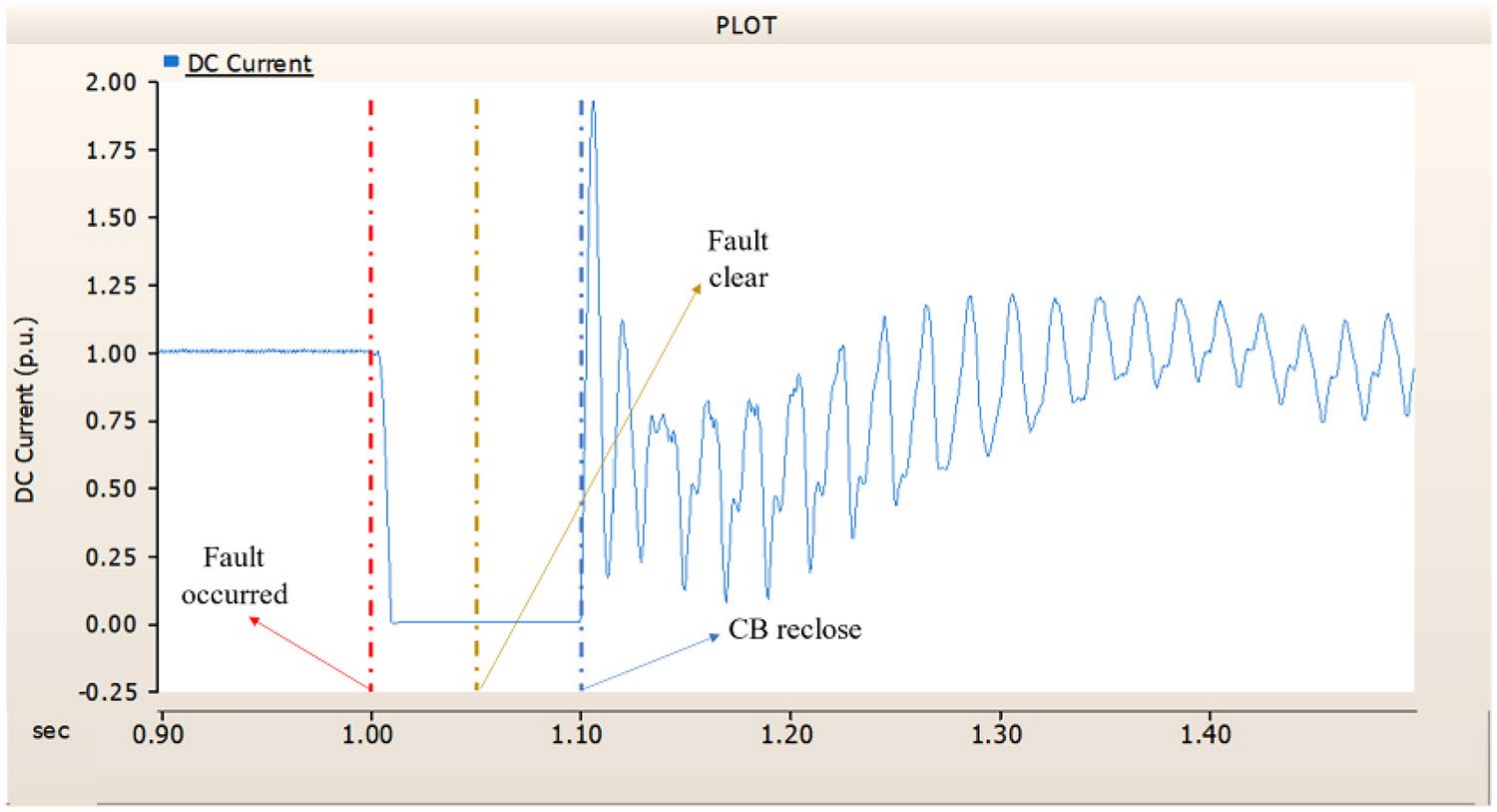
The DC current output pattern of rectifier AC transmission line to ground fault on phase A with CB allocated AC and DC side both.

When an SLG fault occurs in an AC transmission line and circuit breakers are installed on both the DC and AC sides, the transmission side generator appears to recover its original output, as in the case of Sect. 4.1.1.1, but the recovery of the rectifier is very slow. The stability of the DC voltage and current shows a decrease. Considering the power of the transmission generator, this problem seems to be due to the characteristics of the generator regulator. Therefore, there seems to be a need to find ways to improve the controller of the transmission end generator. The transient response of the transmission end generator is shown in Fig. 17, and the transient response of the rectifier is shown in Figs. 18 and 19.

##### Circuit breaker failed to fault clearance

In this chapter, the available failure case of CBs suggested on Table 3, Chap. 4.1.1.2, three cases are conducted. Same as 4.1.1.2, CB operates at 1.00 s. And the failure of CB occurred 0.02 s (i.e., 3 cycles) later.

**Fig. 20:**
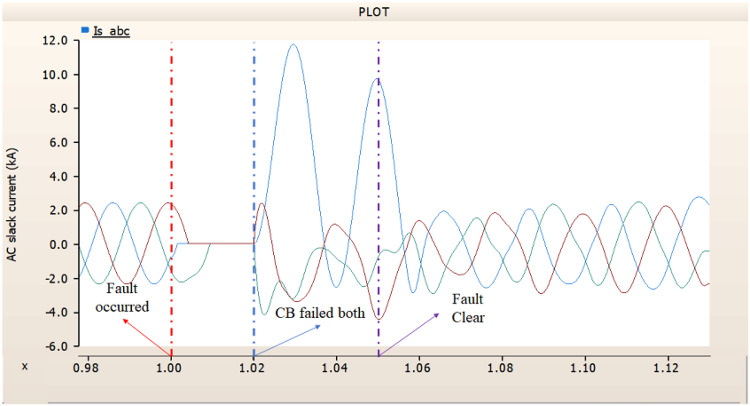
The output pattern of the sending end generator on AC transmission line to ground fault on phase A with CB allocated AC and DC side both. During the fault duration, both the AC side and DC side CB failed.

**Fig. 21:**
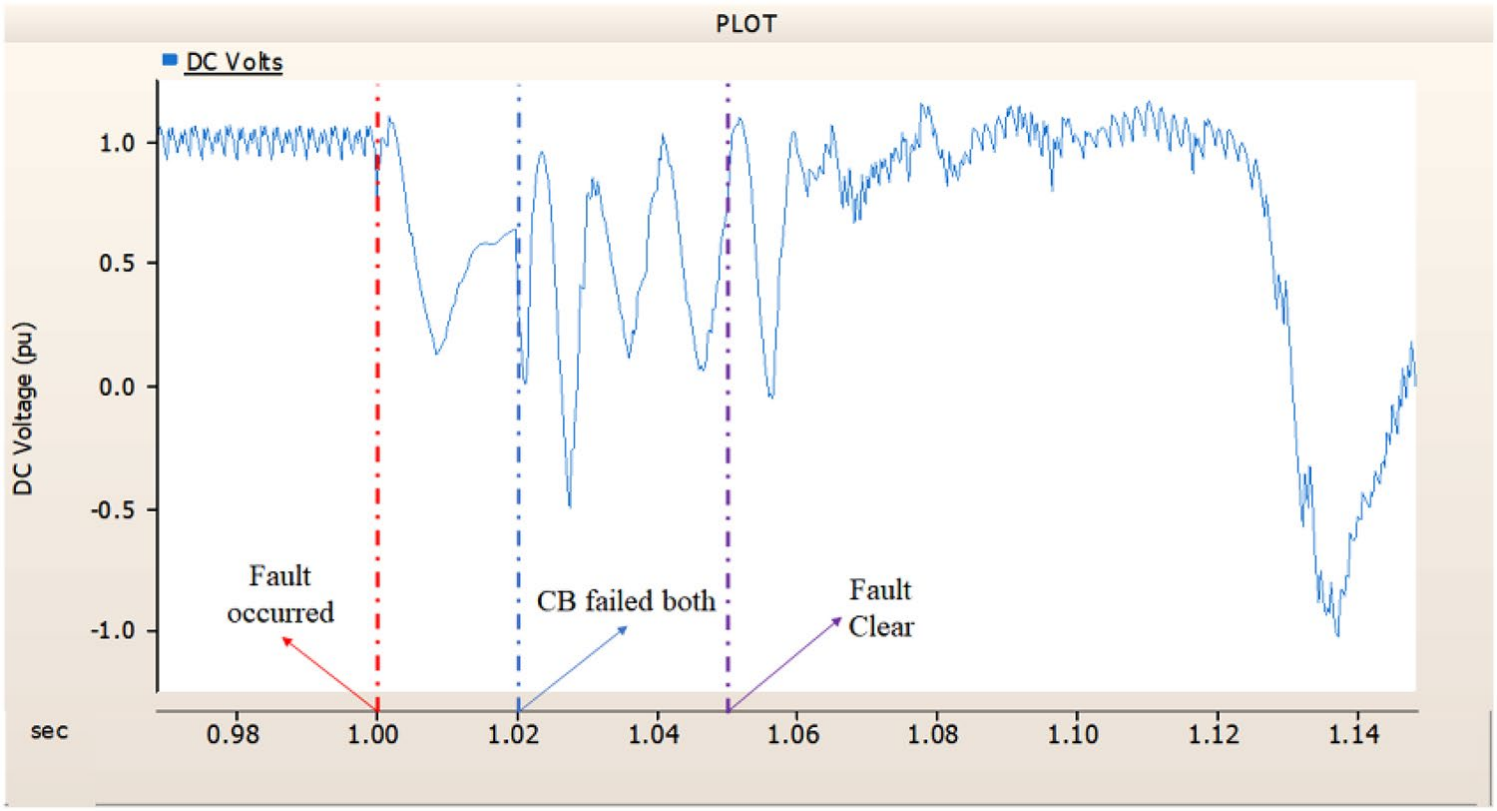
The DC voltage output pattern of rectifier AC transmission line to ground fault on phase A with CB allocated AC and DC side both. During the fault duration, both the AC side and DC side CB failed.

**Fig. 22:**
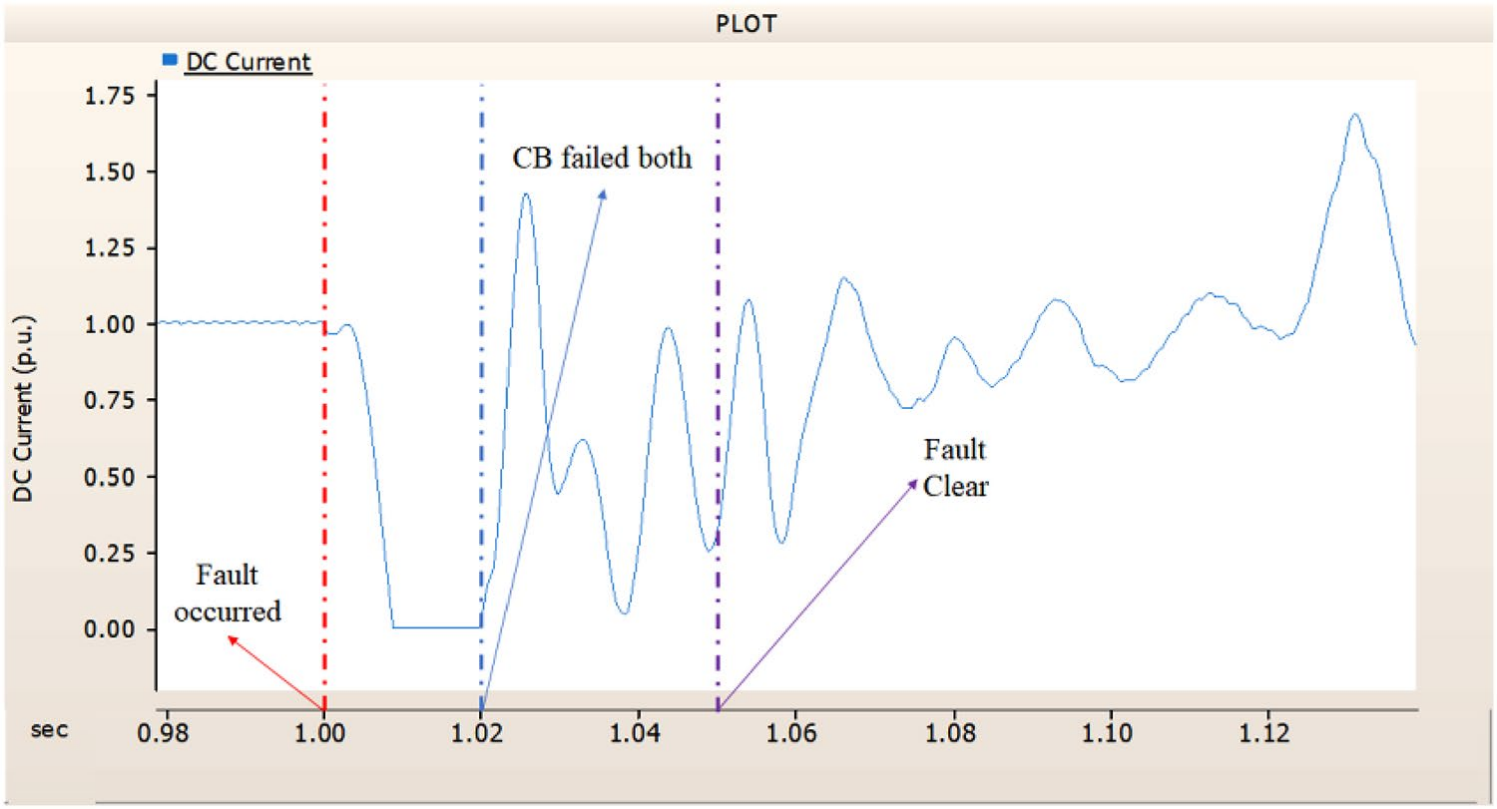
The DC current output pattern of rectifier AC transmission line to ground fault on phase A with CB allocated AC and DC side both. During the fault duration, both the AC side and DC side CB failed.

In the first scenario, as in 4.1.2.1, Fig. 20 ‘s output pattern indicates that the synchronous generator on the transmission side seems to be returning to its initial output. On the other hand, Fig. 20’s peak value is nearly twice as high as Fig. 17’s peak value. Additionally, Figs. 18 and 19 demonstrated the prefault output pattern’s tendency to rebound; Figs. 21 and 22 do not display the pattern. However, based on the DC voltage and DC current output patterns, it appears that if appropriate control strategies are implemented after a fault, it may be possible to recover well.

**Fig. 23:**
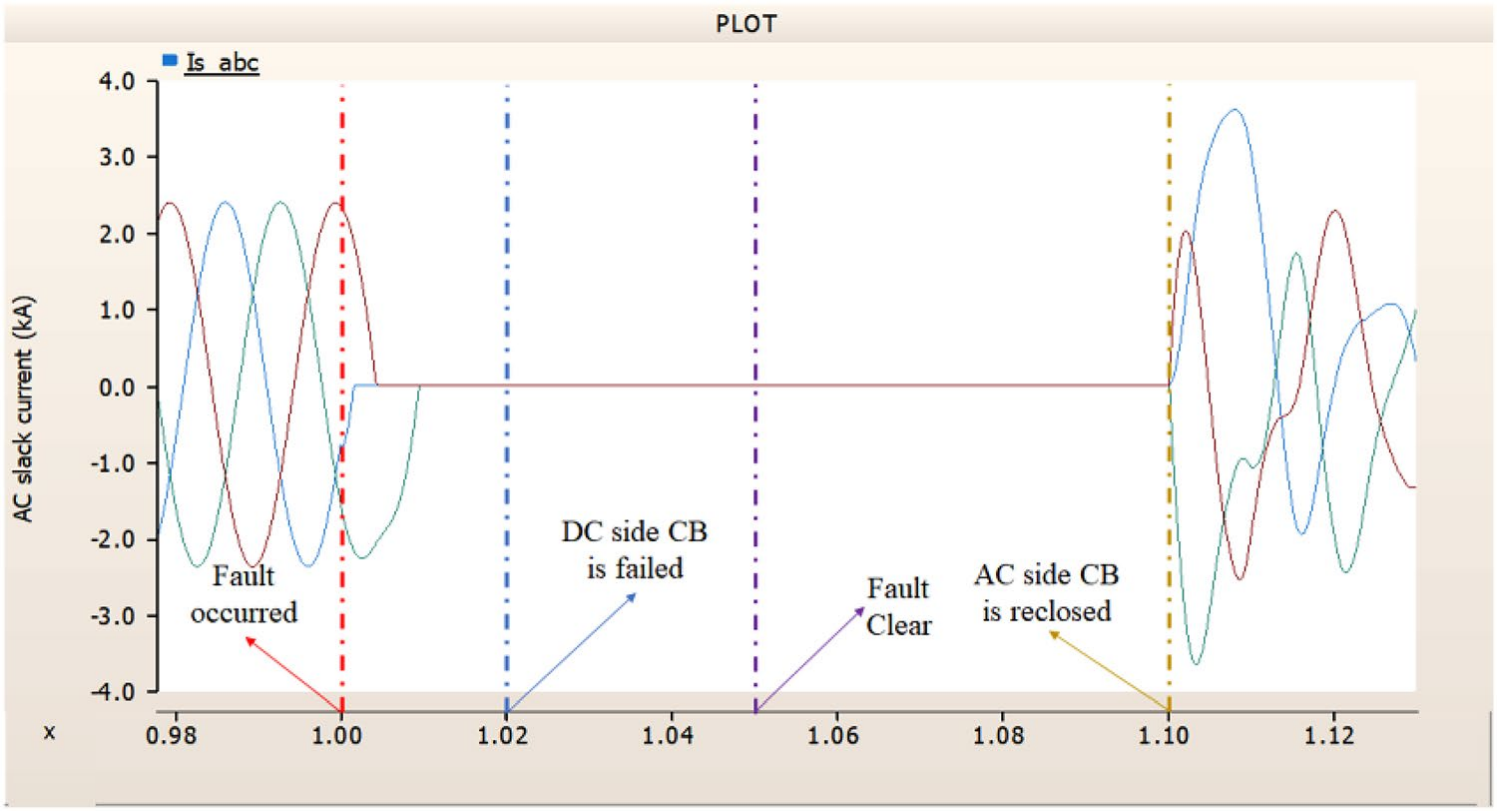
The output pattern of the sending end generator on AC transmission line to ground fault on phase A with CB allocated AC and DC side both. The AC side CB operated well, but the DC side CB failed on fault duration.

**Fig. 24:**
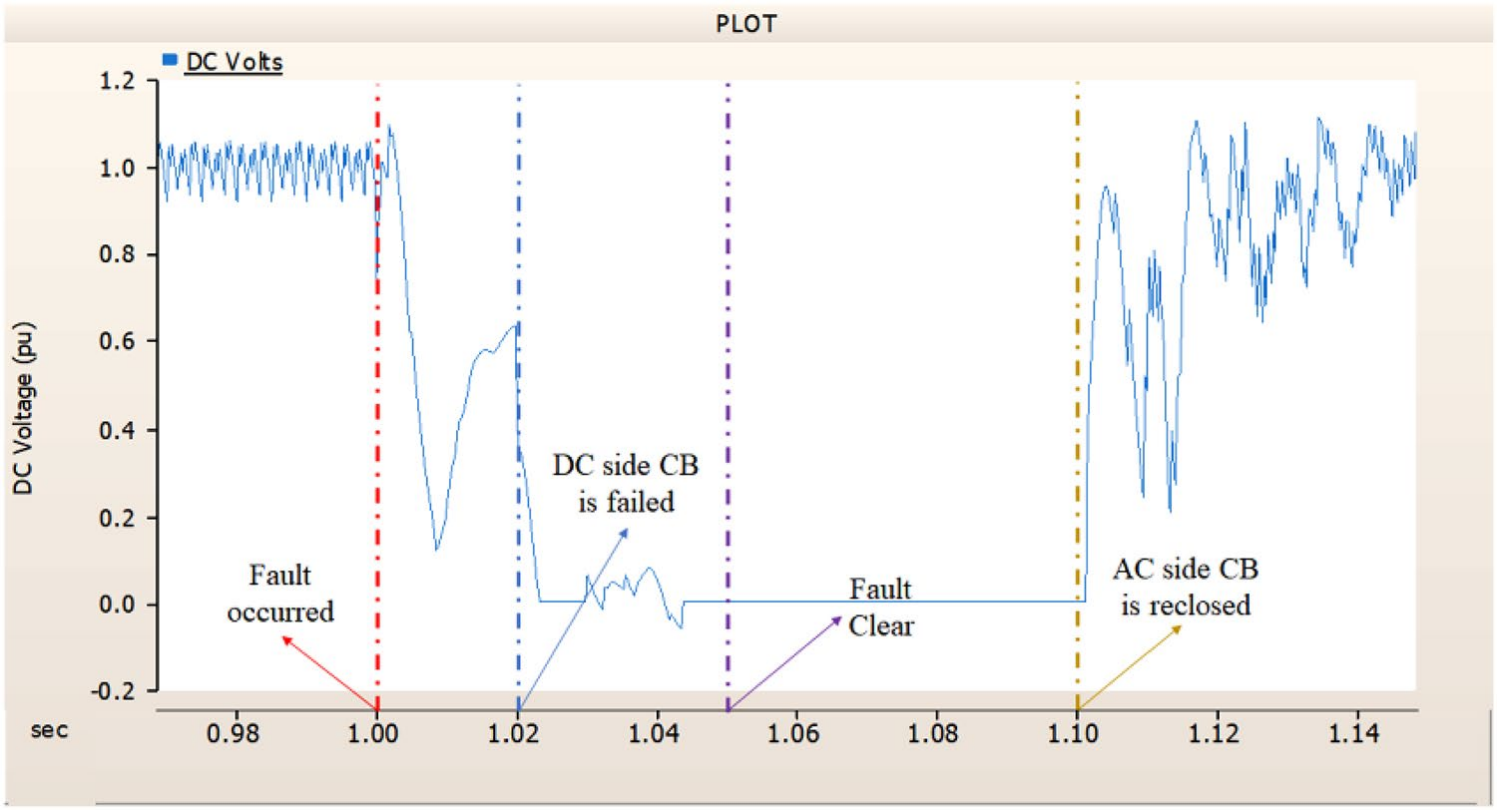
The DC voltage output pattern of rectifier AC transmission line to ground fault on phase A with CB allocated AC and DC side both. The AC side CB operated well, but the DC side CB failed on fault duration.

**Fig. 25:**
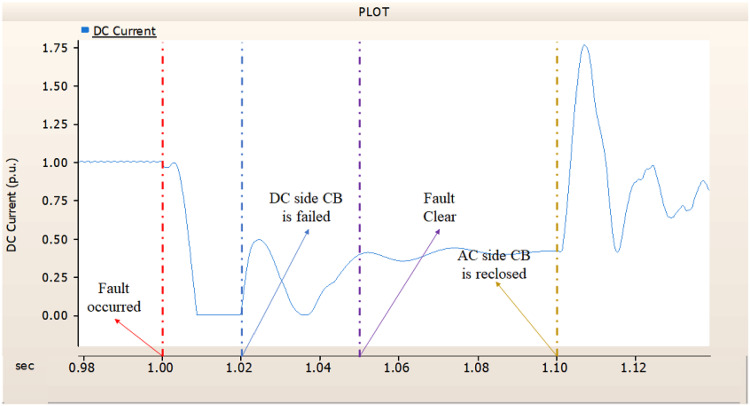
The DC current output pattern of rectifier AC transmission line to ground fault on phase A with CB allocated AC and DC side both. The AC side CB operated well, but the DC side CB failed on fault duration.

In the second scenario, the output pattern of generator and rectifier illustrated in Figs. 23, 24 and 25 shows the tendency to recover its original outputs as in the case of 4.1.2.1. Considering the DC voltage and DC current of the rectifier illustrated in Figs. 24 and 25, the outputs can be recovered well if the control strategies of generator and rectifier together.

**Fig. 26:**
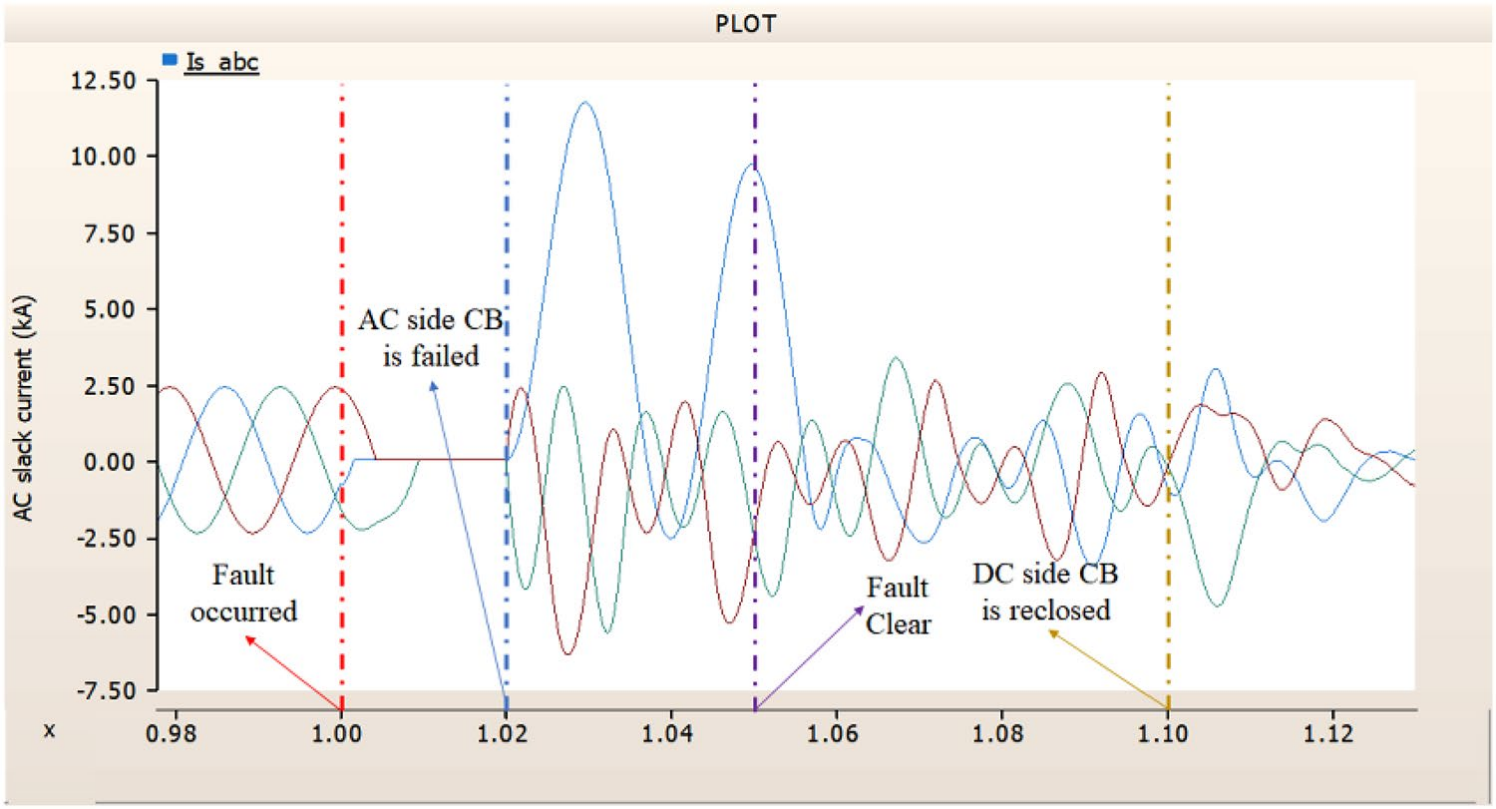
The output pattern of the sending end generator on AC transmission line to ground fault on phase A with CB allocated AC and DC side both. The DC side CB operated well, but the AC side CB failed on fault duration.

**Fig. 27:**
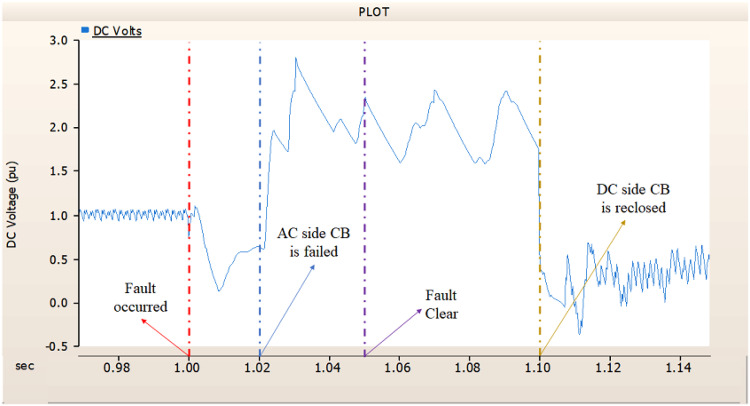
The DC voltage output pattern of rectifier AC transmission line to ground fault on phase A with CB allocated AC and DC side both. The DC side CB operated well, but the AC side CB failed on fault duration.

**Fig. 28:**
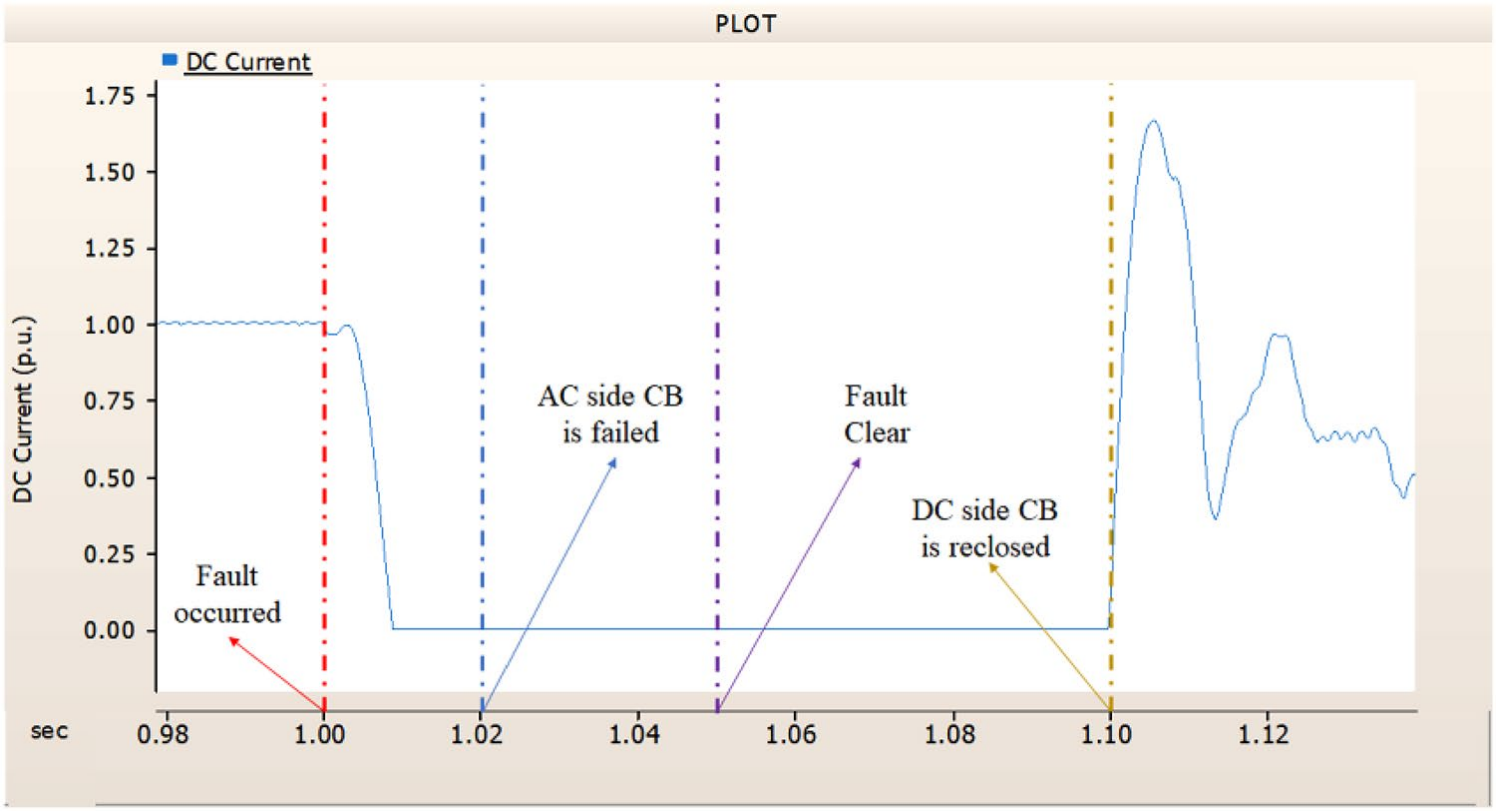
The DC current output pattern of rectifier AC transmission line to ground fault on phase A with CB allocated AC and DC side both. The AC side CB operated well, but the DC side CB failed on fault duration.

In the third scenario, considering the operation of CBs, the output pattern of rectifier illustrated in Figs. 27 and 28 is derived from synchronous generator. In Fig. 26, the AC side CB is failed to operation during fault, the generator’s current output on phase A relatively very large compared with phase B and C.

### CB is only on the AC side

#### DC transmission line-to-ground fault

##### Circuit breaker operated well on fault duration

**Fig. 29:**
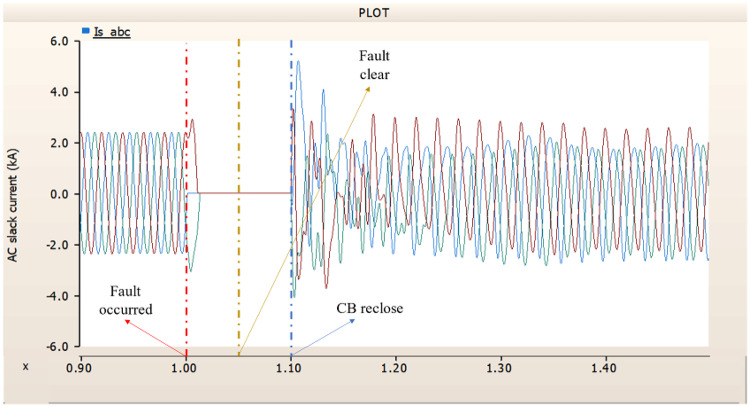
The output pattern of sending side generator DC transmission line-to-ground fault with CB allocated only on the AC side.

**Fig. 30:**
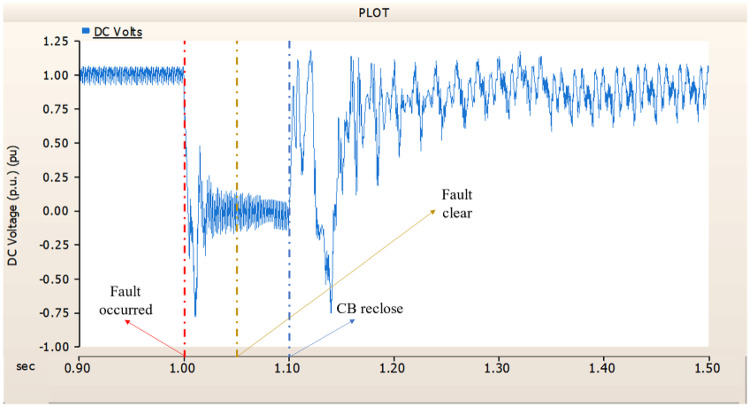
The DC voltage output pattern of rectifier DC transmission line-to-ground fault with CB allocated only on the AC side.

**Fig. 31:**
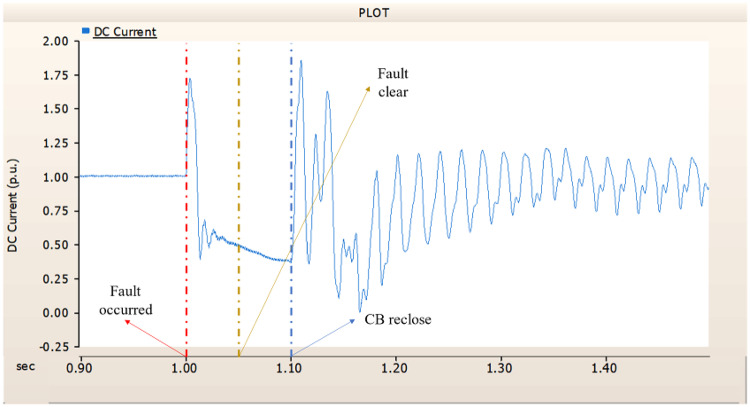
The DC current output pattern of rectifier DC transmission line-to-ground fault with CB allocated only on the AC side.

When a circuit breaker is installed solely on the AC side and a ground fault occurs in a DC transmission line, the output appears to be restored before the failure, but the peak value is not recovered. As mentioned above, it is believed to be caused by the characteristics of the generator’s controller. Considering the output pattern of the transient state shown in Figs. 29, 30 and 31, it appears necessary to find a way to simultaneously improve the controller on the rectifier side and the governor or exciter of the synchronous generator at the transmission side.

##### Circuit breaker failed to fault clearance

**Fig. 32:**
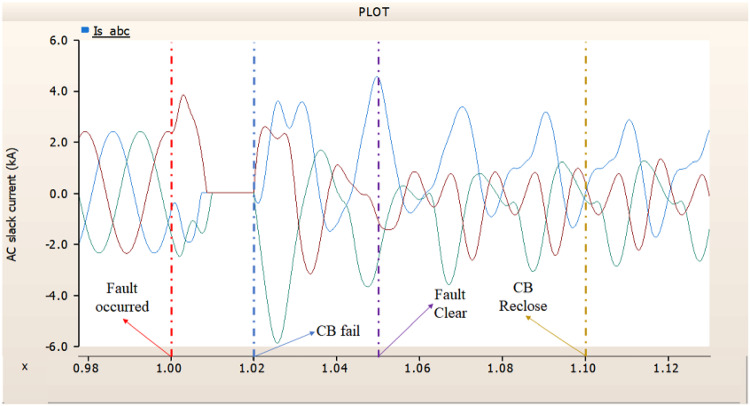
The output pattern of sending side generator DC transmission line-to-ground fault with CB allocated only on the AC side; CB is failed on fault duration.

**Fig. 33:**
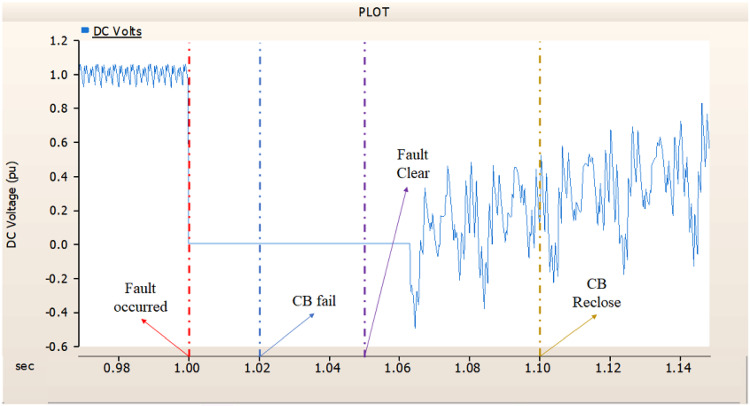
The DC voltage output pattern of rectifier DC transmission line-to-ground fault with CB allocated only on the AC side; CB is failed on fault duration.

**Fig. 34:**
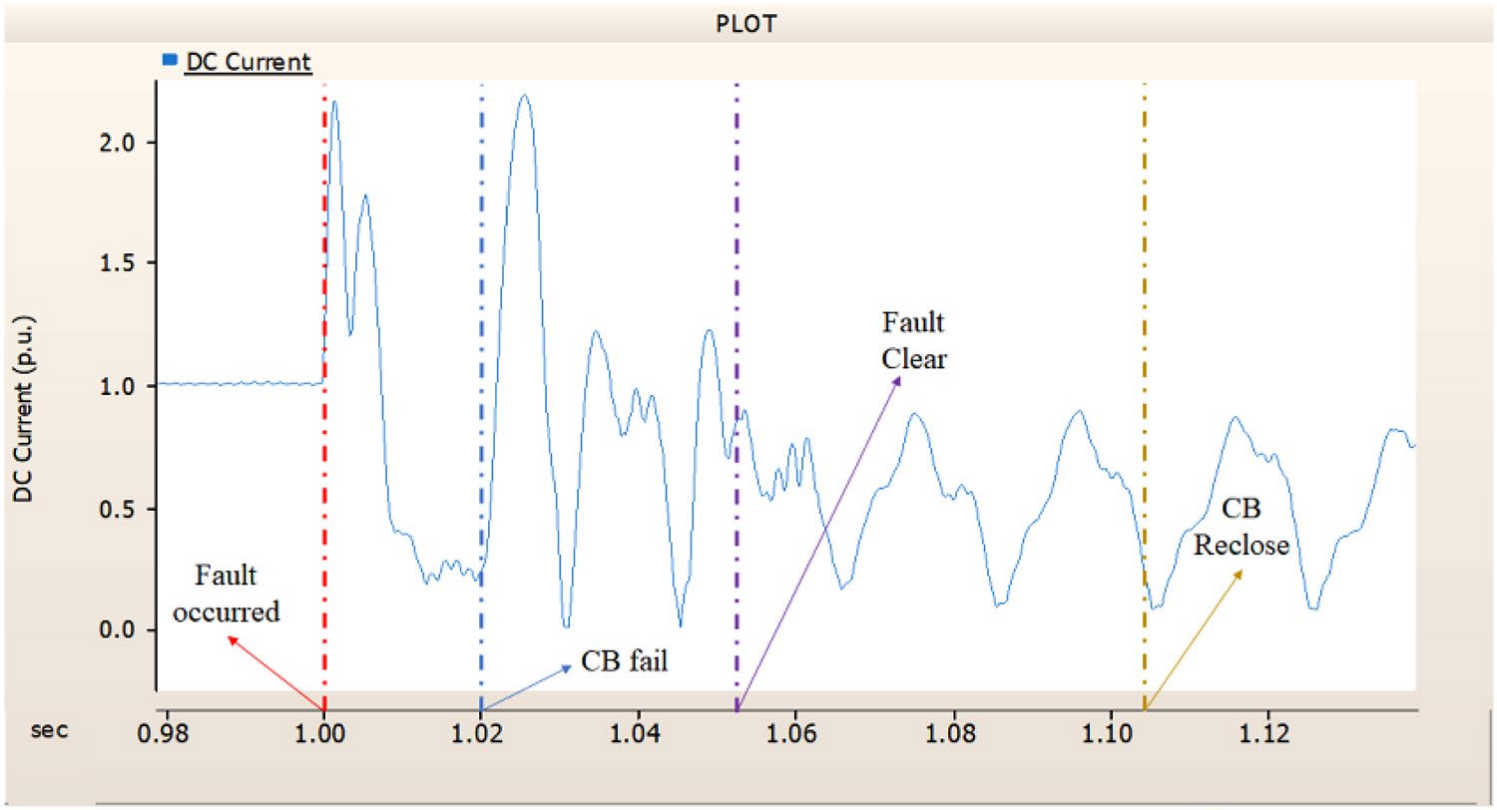
The DC current output pattern of rectifier DC transmission line-to-ground fault with CB allocated only on the AC side; CB is failed on fault duration.

This section simulates an DC transmission line-to-ground fault on DC transmission line when there is just an AC side circuit breaker installed. Furthermore, the circuit breaker is explicitly allocated on the DC side and trips in 1.00 s, and fails on three cycles (i.e., 1.02 s). Figure 32 displays the deformed sending side synchronous generator output pattern as a result of the CB’s operation failure. Furthermore, Figs. 33 and 34 demonstrate the inability to revert to the prefault output pattern, in contrast to 4.1.1.1. Finding a means to simultaneously enhance the synchronous generator at the transmission side and the rectifier side controller at the after fault clearing stage is crucial, given the results displayed in Figs. 32, 33 and 34.

#### AC transmission line SLG fault on phase A

##### Circuit breaker operated on fault duration

**Fig. 35:**
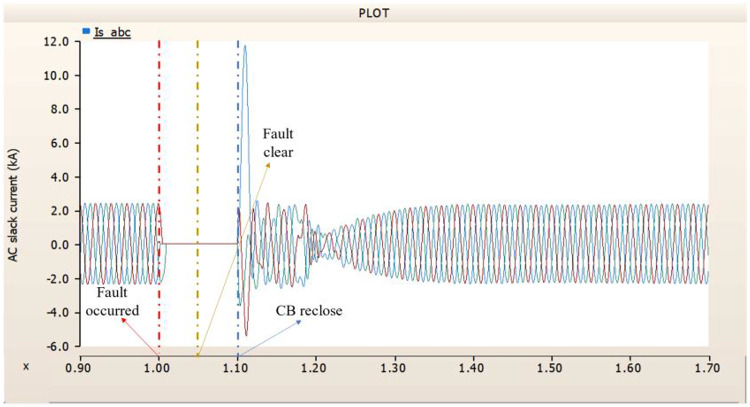
The output pattern of sending side end generator AC transmission line to ground fault on phase A with CB allocated only on the AC side.

**Fig. 36:**
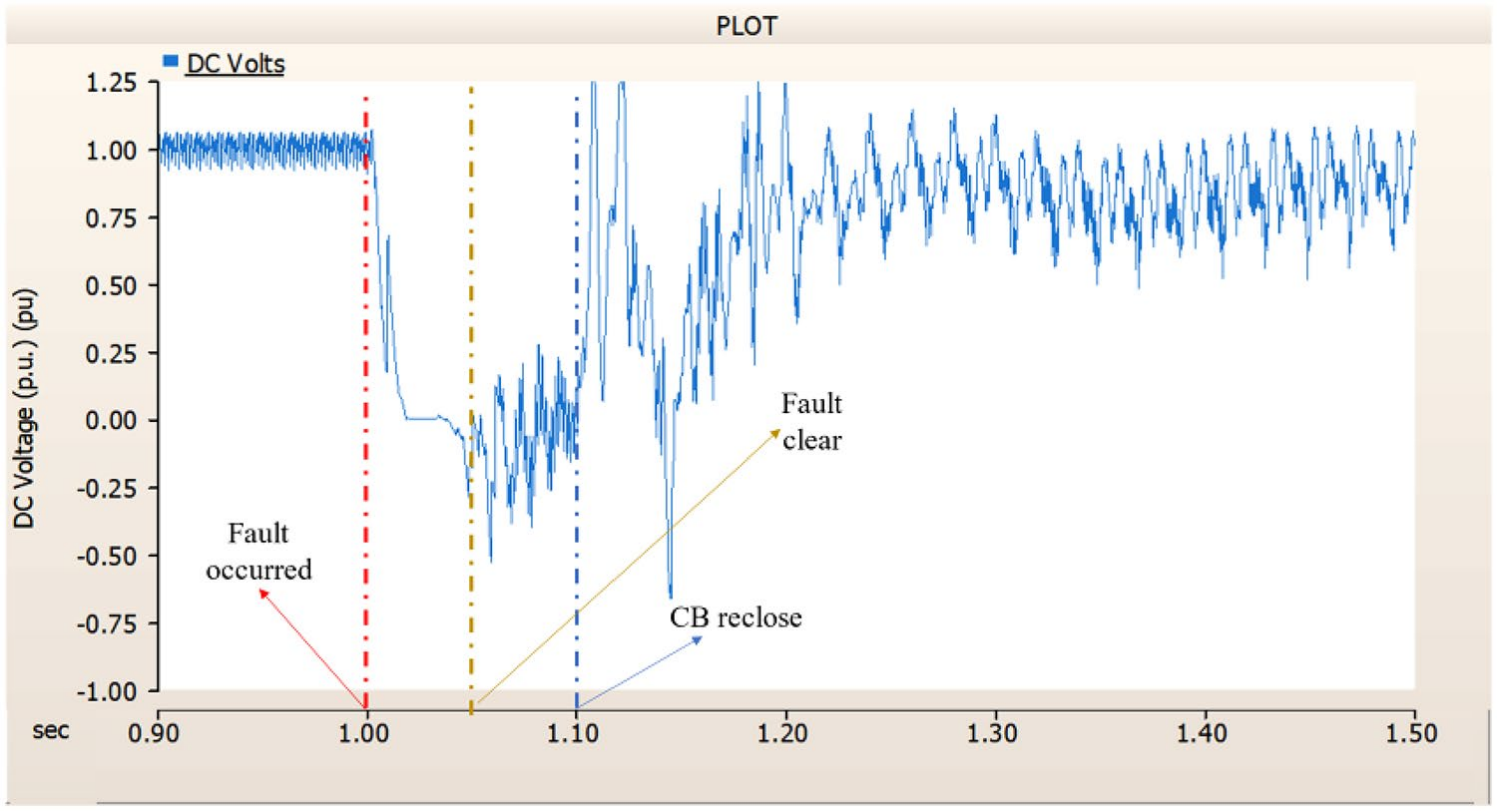
The DC voltage output pattern of rectifier AC transmission line to ground fault on phase A with CB allocated only on the AC side.

**Fig. 37:**
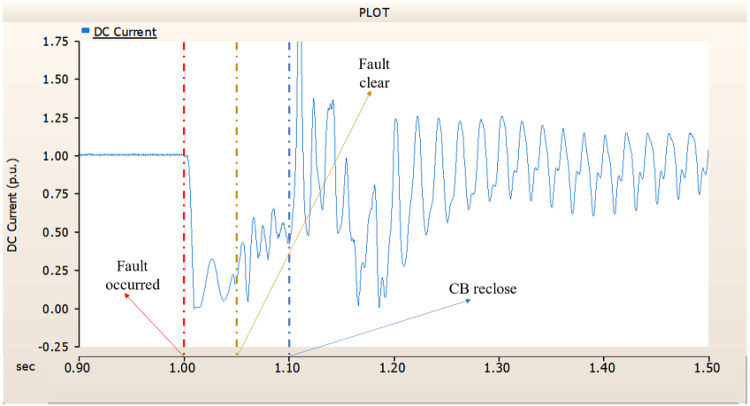
The DC current output pattern of rectifier AC transmission line to ground fault on phase A with CB allocated only on the AC side.

When a ground fault accident in an AC transmission line is simulated in a situation where a circuit breaker is installed only on the AC side, the transmission stage generator shows a tendency to recover to the prefault state as shown in Fig. 35, but in the case of the rectifier, Fig. 36. As shown in Fig. 37, it shows a tendency not to recover to the original output pattern. Therefore, if a circuit breaker is placed only on the AC side, it is necessary to find ways to improve the rectifier control.

##### Circuit breaker failed to fault clearance

**Fig. 38:**
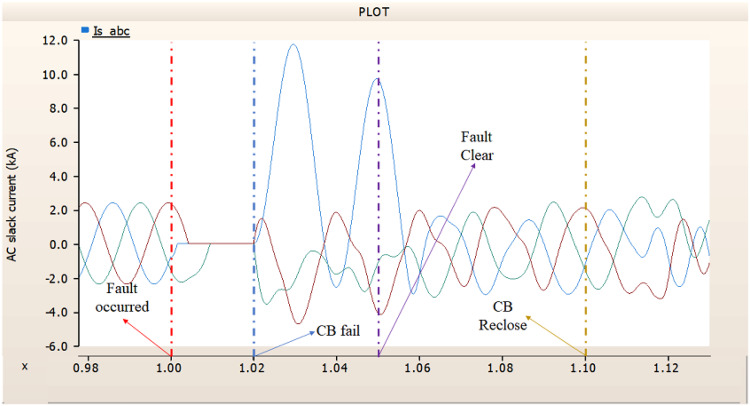
The output pattern of sending side end generator AC transmission line to ground fault on phase A with CB allocated only on the AC side; CB is failed on fault duration.

**Fig. 39:**
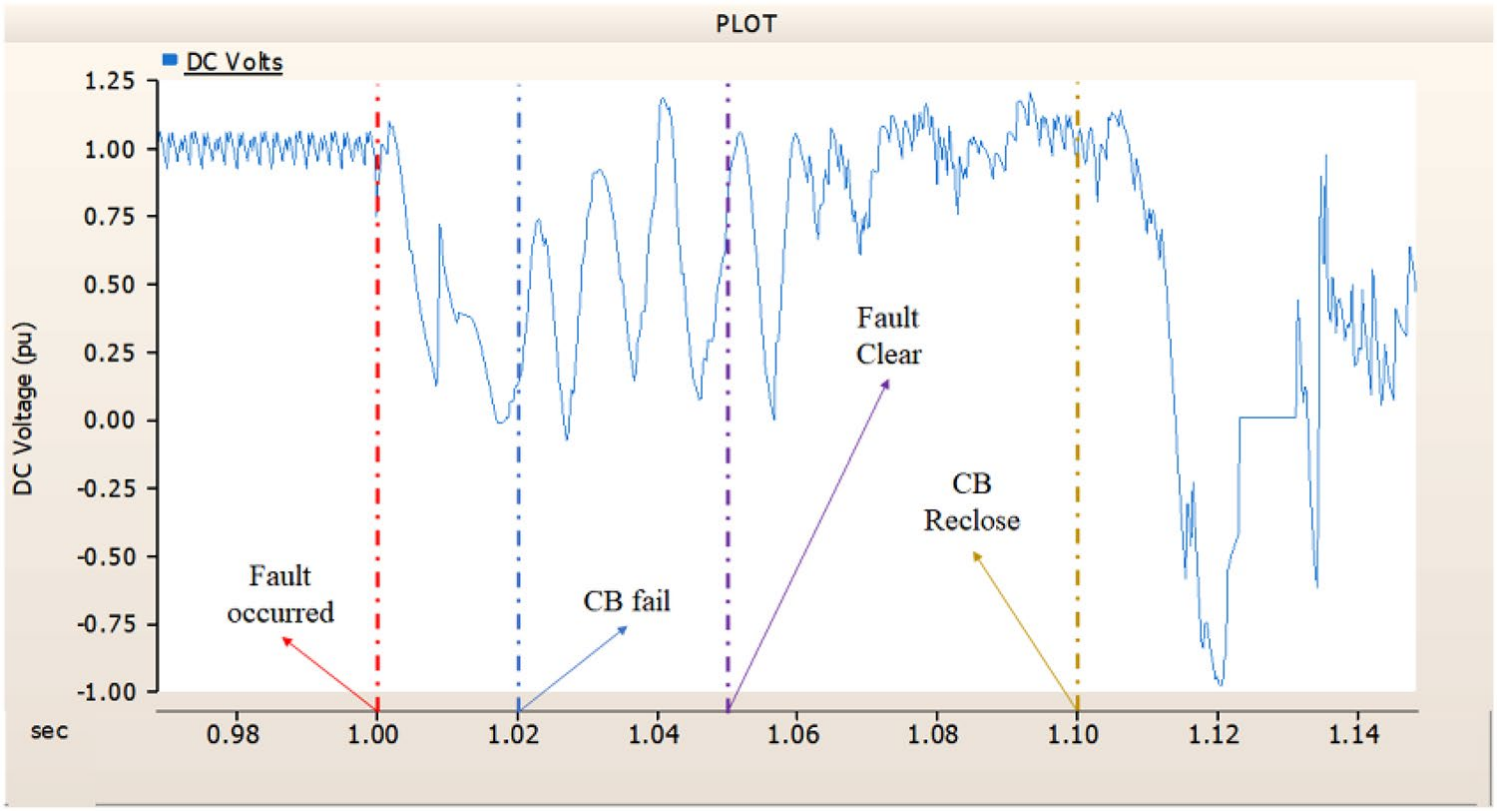
The DC voltage output pattern of rectifier AC transmission line to ground fault on phase A with CB allocated only on the AC side; CB is failed on fault duration.

**Fig. 40:**
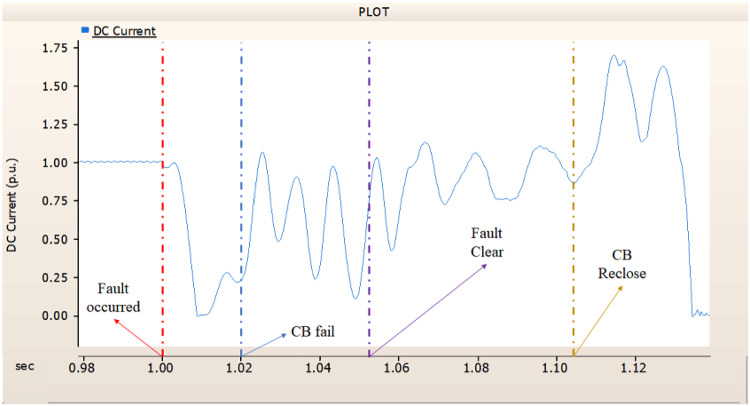
The DC current output pattern of rectifier AC transmission line to ground fault on phase A with CB allocated only on the AC side; CB is failed on fault duration.

This section simulates an SLG fault on phase A in an AC transmission line when there is just an AC side circuit breaker installed. Furthermore, the circuit breaker is explicitly allocated on the DC side and trips in 1.00 s, and fails on three cycles (i.e., 1.02 s). Figure 38 illustrates the transmission stage generator’s propensity to return to the prefault state. However, as Figs. 39 and 40 demonstrate, the rectifier exhibits a propensity to deviate from the initial output pattern. Furthermore, the phenomena illustrates the transfer of reverse power when looking at Fig. 39 ‘s output pattern. Therefore, it is vital to figure out how to enhance the rectifier control if the circuit breaker is just located on the AC side.

### CB is only on the DC side

#### DC transmission line-to-ground fault

##### Circuit breaker operated well on fault duration

**Fig. 41:**
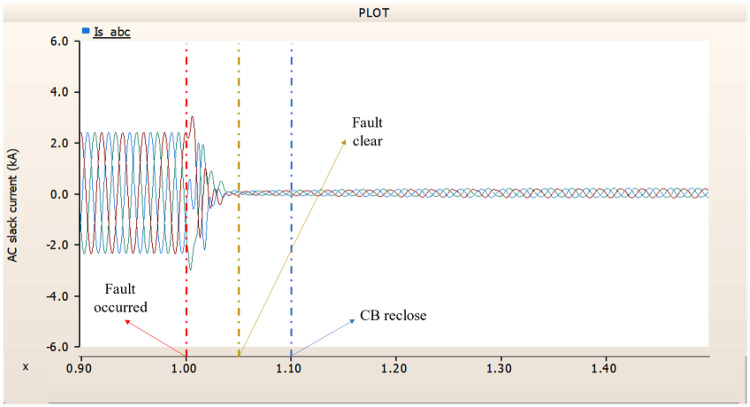
The output pattern of sending side generator DC transmission line-to-ground fault with CB allocated only on the DC side.

When simulating a line-to-ground fault on a DC transmission line, assuming a scenario where a circuit breaker is exclusively assigned to the DC side and operates effectively throughout the fault duration, both the generator and the rectifier on the transmission side show a problem of not being able to recover their original output. In particular, considering both the output pattern of the transmission side generator and the out pattern of the rectifier shown in Fig. 41-Fig. 43, not only is the original output value not restored, but reverse transmission is also occurs. Looking at the controller output of the rectifier and inverter, control of the firing angle and overlap angle occurs to maintain stability and reconnection from the time of failure to the time of reconnection. This can be confirmed by Figs. 44 and 45. However, as shown in Fig. 45, the overlap angle becomes zero, and in the process of controlling this, a commutation failure occurs, which prevents power conversion from DC to AC at the inverter stage and causes one-way transmission to not occur properly. This phenomenon is due to the characteristics of the controller. The controller consists of three levels: top, middle, and bottom. At present, the top controller is responsible for system management and controlling the frequency and active power flow. The middle controller is responsible for controlling DC current, voltage, and grid voltage unbalance, and the bottom controller is responsible for gate pulse generation, phase lock oscillator, and gate pulse timing^35^. Specifically, the top controller at the transmitting end controls the DC current, and the top controller at the receiving end controls the DC voltage. At this time, if a fault occurs, the DC voltage at the receiving end becomes zero and acts as a disturbance to the controller. The top-level controller uses a proportional-integral controller. Due to the characteristics of such a controller, control is performed by proportional and integral error, but when the DC voltage becomes zero, the value of the controllable disturbance is exceeded. These control characteristics suggest that it is inappropriate to establish a strategy for placing circuit breakers only on the DC side in the event of a ground fault on a DC transmission line.

**Fig. 42:**
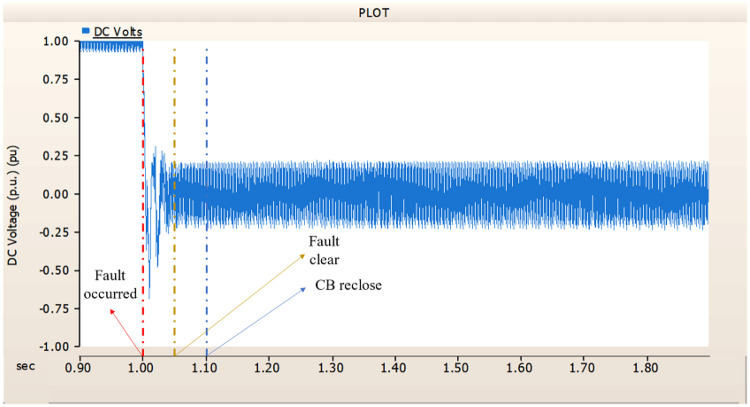
The DC voltage output pattern of rectifier DC transmission line-to-ground fault with CB allocated only on the DC side.

**Fig. 43:**
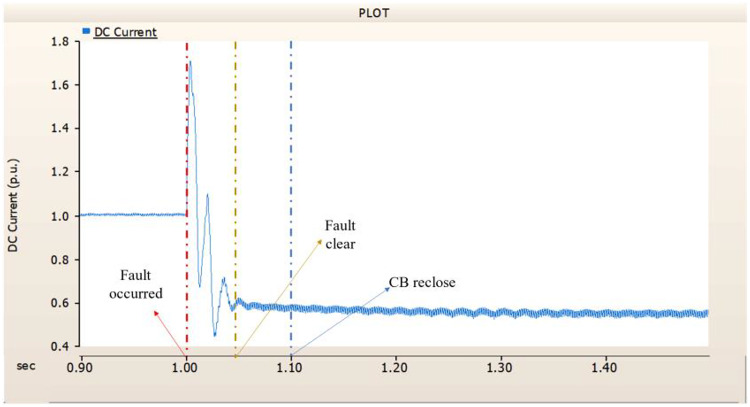
The DC current output pattern of rectifier DC transmission line-to-ground fault with CB allocated only on the DC side.

**Fig. 44:**
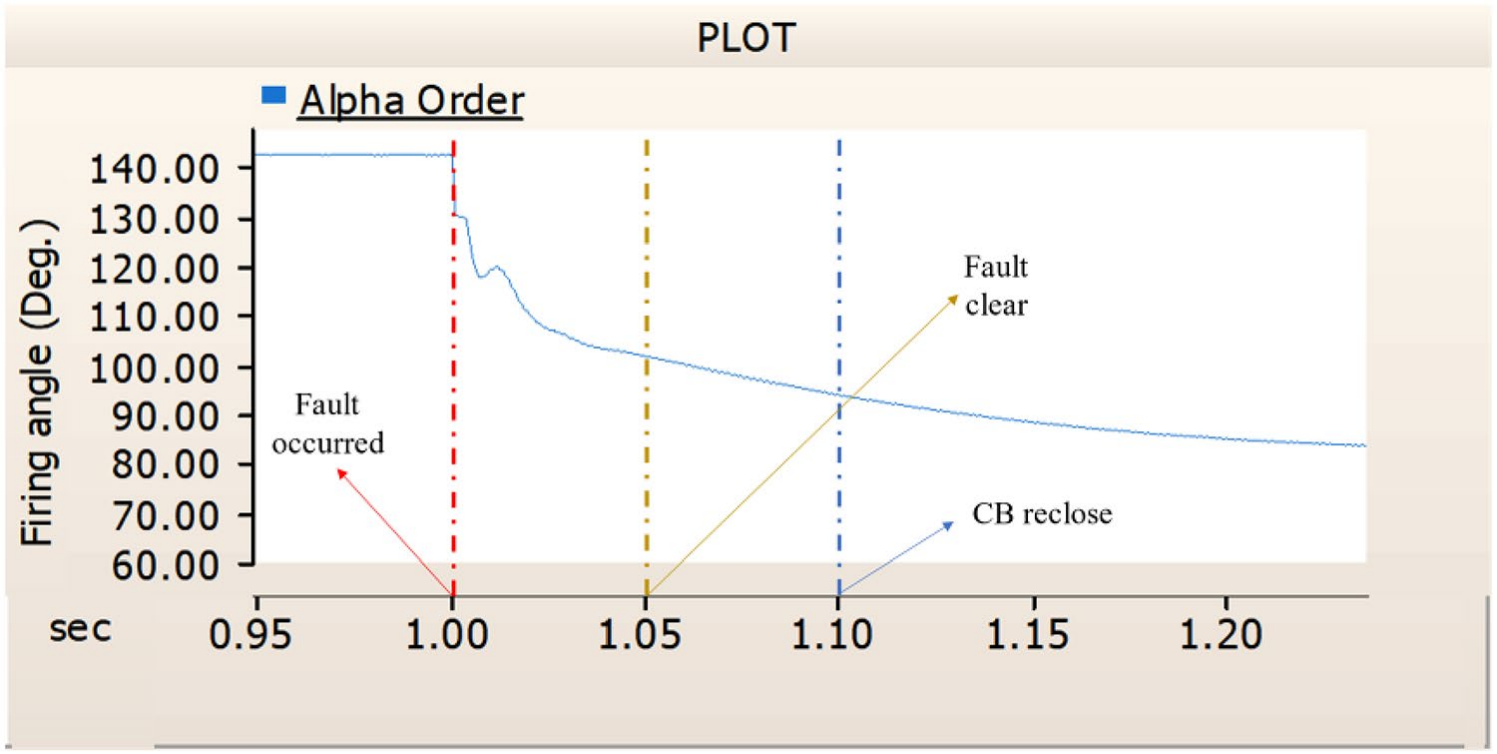
The rectifier’s firing angle output pattern of rectifier controller DC transmission line-to-ground fault with CB allocated only on the DC side.

**Fig. 45:**
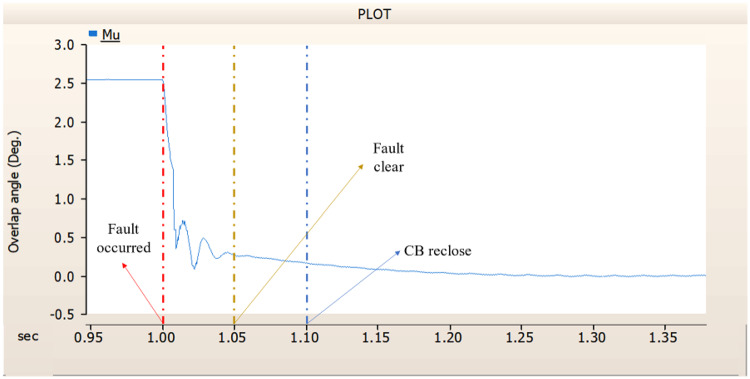
The rectifier’s output pattern of overlap angle DC transmission line-to-ground fault with CB allocated only on the DC side.

##### Circuit breaker failed to fault clearance

**Fig. 46:**
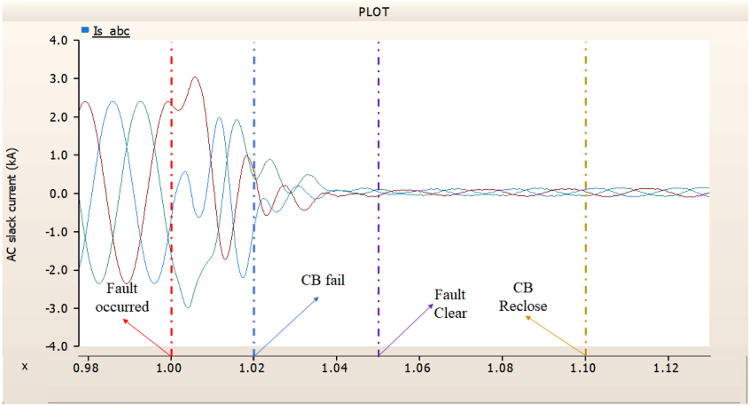
The output pattern of a sending side generator DC transmission line-to-ground fault with CB allocated only on the DC side; CB is failed on fault duration.

**Fig. 47:**
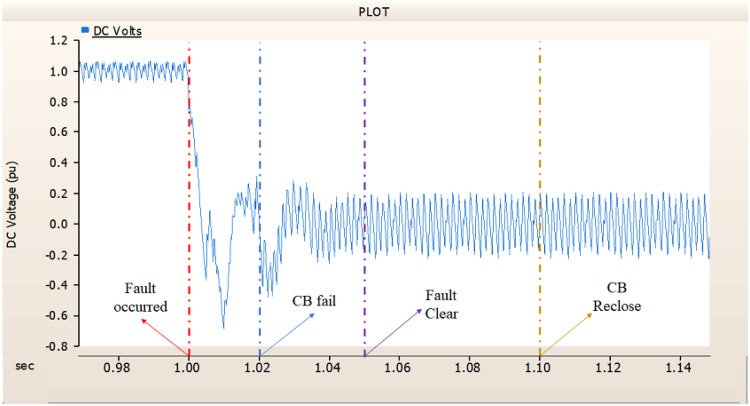
The DC voltage output pattern of a rectifier DC transmission line-to-ground fault with CB allocated only on the DC side; CB is failed on fault duration.

**Fig. 48:**
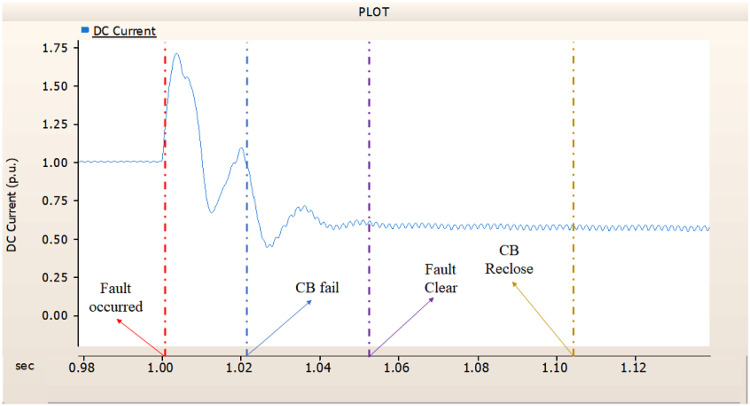
The DC current output pattern of a rectifier DC transmission line-to-ground fault with CB allocated only on the DC side; CB is failed on fault duration.

**Fig. 49:**
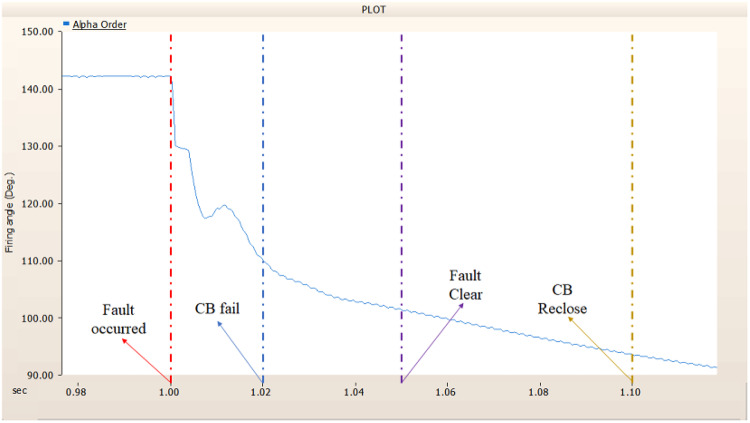
The firing angle output pattern of the rectifier controller in a DC transmission line-to-ground fault with CB allocated only on the DC side; CB is failed on fault duration.

**Fig. 50:**
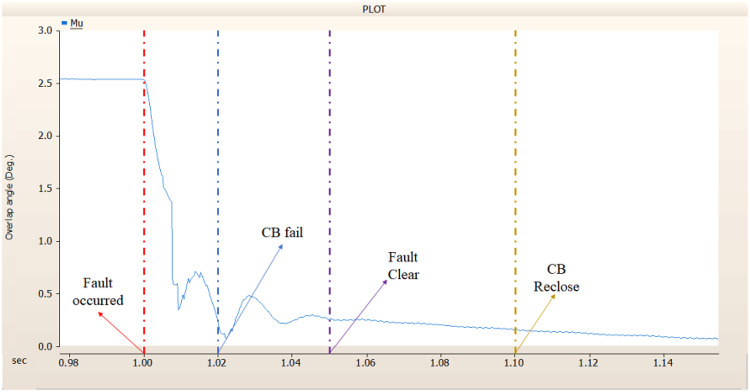
The output pattern of the rectifier’s overlap angle DC transmission line-to-ground fault with CB allocated only on the DC side; CB is failed on fault duration.

During the simulation of a line-to-ground fault on a DC transmission line, if a circuit breaker is specifically designated to the DC side and fails within 1.02 s (i.e., 3 cycles), both the generator and the rectifier on the transmission side are unable to return to their original output. Specifically, taking into account the output patterns of the rectifier and the transmission side generator as illustrated in Figs. 46, 47 and 48, which correspond to 4.3.1.1, it can be observed that not only is the initial output value lost, but reverse transmission also takes place. Also, the current value of generator of transmission side becomes almost zero. Similar to 4.3.1.1, as seen in Figs. 49 and 50, the overlap angle decreases to zero and, while attempting to manage this, a commutation failure takes place. This stops power conversion from DC to AC at the inverter stage and results in improper one-way transmission. These control features imply that, in the event of a ground fault on a DC transmission line, it is not acceptable to devise a plan for exclusively deploying circuit breakers on the DC side on both case; CB operated successfully or failed to fault clearance.

#### AC transmission line SLG fault on phase A

##### Circuit breaker operated well on fault duration

When the SLG fault in a DC transmission line is simulated after installing a single circuit breaker on the AC side, both the generator and the rectifier on the transmission side show a tendency to return to the original output pattern, but they do not completely return to the original pattern. However, unlike Sect. 4.3.1, due to the characteristics of the controller described above, when both the AC and DC sides are blocked, the same disturbance occurs in both the DC current control and the DC voltage control, so the error is within the range that the proportional-integral controller can handle. It appears to recover to a certain level after the fault is removed. As shown inFigure **51**, the generator at the transmission end is unable to recover the peak value. As shown in Fig. 52 and Figure **53**, the voltage does not recover its peak value and the current shows very large fluctuations. First of all, it is suggested to adjust the regulator of the exciter/governor of the synchronous generator at the transmission end and the regulator of the rectifier in parallel, as in Sect. 4.2.1.

**Fig. 51:**
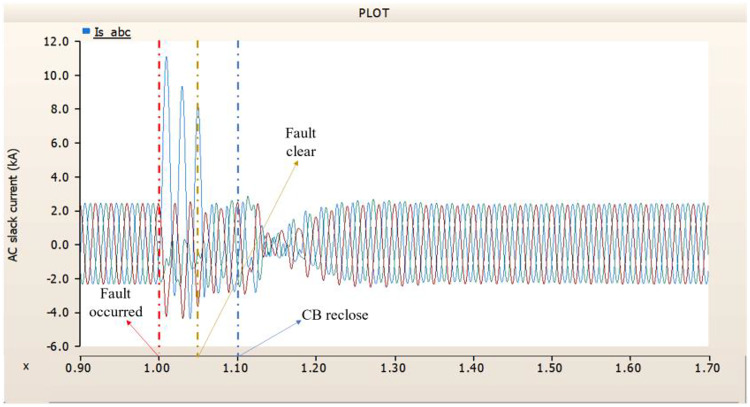
The output pattern of sending side generator AC transmission line to ground fault on phase A with CB allocated only on the DC side.

**Fig. 52:**
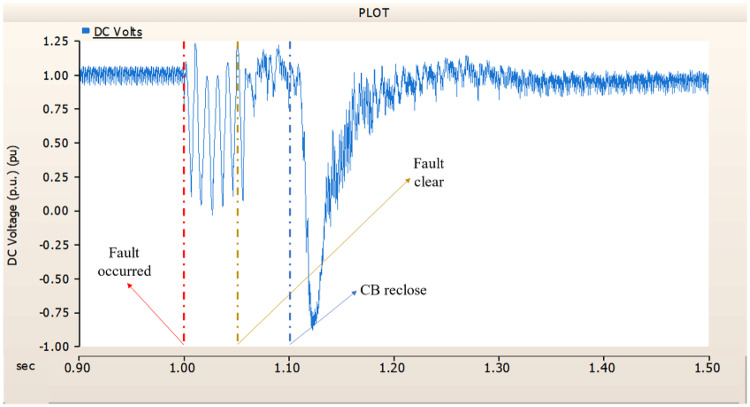
The DC voltage output pattern of rectifier AC transmission line to ground fault on phase A with CB allocated only on the DC side.

**Fig. 53:**
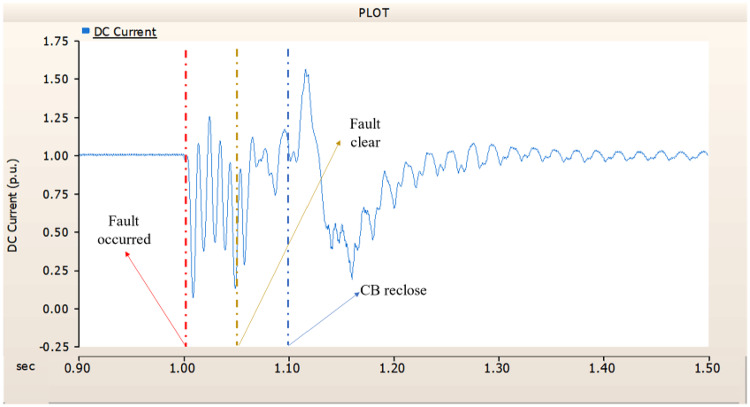
The DC current output pattern of rectifier AC transmission line to ground fault on phase A with CB allocated only on the DC side.

##### Circuit breaker failed to fault clearance

**Fig. 54:**
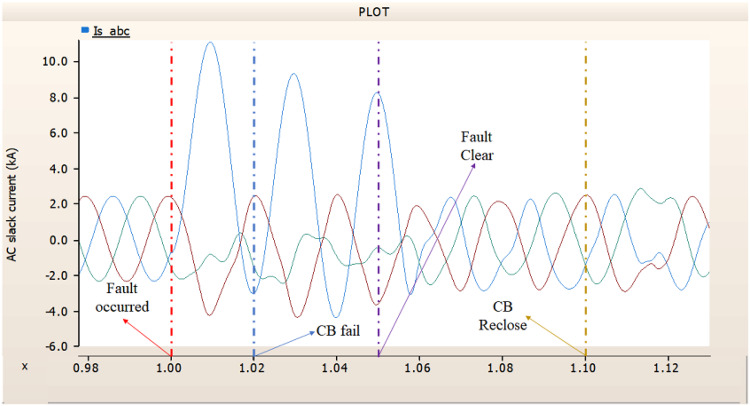
The output pattern of sending side generator DC transmission line-to-ground fault with CB allocated only on the AC side; CB is failed on fault duration.

**Fig. 55:**
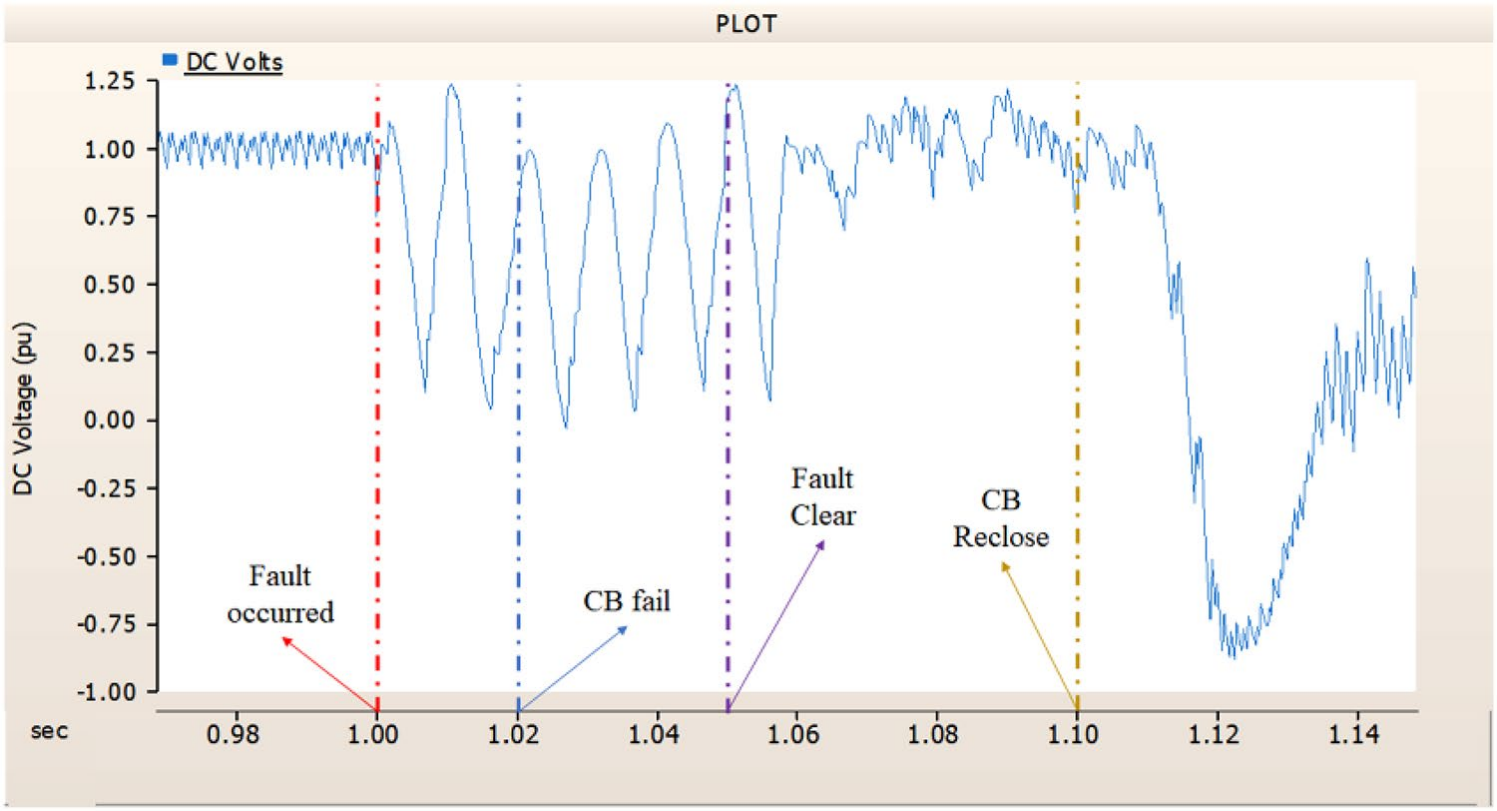
The DC voltage output pattern of rectifier DC transmission line-to-ground fault with CB allocated only on the AC side; CB is failed on fault duration.

**Fig. 56:**
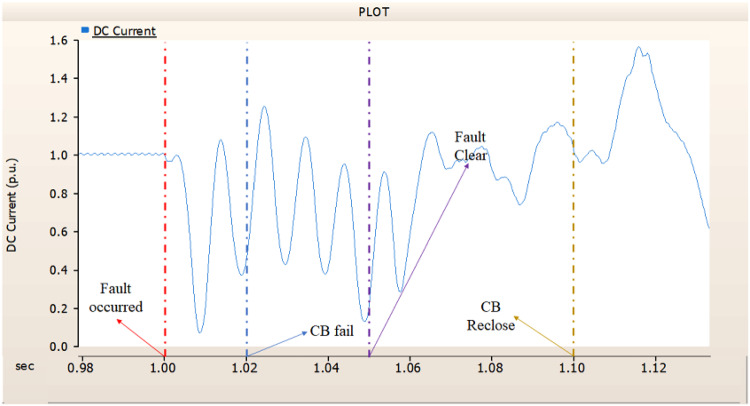
The DC current output pattern of rectifier DC transmission line-to-ground fault with CB allocated only on the AC side; CB is failed on fault duration.

Also, same as previous cases, circuit breaker is specifically designated to the DC side and fails within 1.02 s (i.e., 3 cycles). In contrast to 4.3.2.1, both the generator and the rectifier on the transmission side shown a tendency to be unable to recover to the original output pattern when a circuit breaker is installed solely on the AC side and a ground fault occurs in a DC transmission line. In contrast to 4.3.2.1, as shown in Fig. 54, the generator at the transmission end became as zero. Furthermore, as Figs. 55 and 56 demonstrate, the output pattern of DC voltage and DC current is unable to revert to its initial state. In particular, the transmission system exhibits reverse transmission when examining Fig. 55’s output pattern. That is, in contrast to 4.3.2.1, the proportional-integral controller is unable to tolerate faults in the control of DC voltage and DC current when the CB fails.

## Conclusion

In this study, the dynamic characteristics of the system were simulated using PSCAD/EMTDC to consider the allocation and operation plan of circuit breakers taking into account the transient response of the system. In particular, it is encouraging that a case study was conducted to consider the layout of circuit breakers that also take into account their failure to trip. As a result of checking the dynamic transient response of the system through simple protection relay settings for failures in AC and DC transmission lines, the case of Sect. 4.3.1 was confirmed that not only the original output value was not restored, but even reverse transmission occurred. In addition, the results of all case studies generally showed that when the circuit breaker was reclosed after a fault was cleared, it did not return to its original output pattern. This study confirmed that it is inappropriate to place a circuit breaker on the DC side when a DC transmission line accident is assumed at the transmission end. In addition, it was confirmed that the robustness of the controller needs to be improved and adapted in order to restart the transmission system after performing protection cooperation by placing circuit breakers. Considering the transient response of the system, the main operation is to install a circuit breaker on the DC side in the event of an AC transmission line accident at the transmission end, but based on a method of installing circuit breakers on both the DC and AC sides in some sections where stable operation of the system is required. In addition, it was confirmed that in all cases when a blocking failure occurs in the circuit breaker, the original output pattern cannot be restored, so it was confirmed that the development of a control technique that takes this into account is also necessary. It seems that cooperation and protection tactics ought to be applied. Furthermore, it appears that the controller must be modified to guarantee quick recovery and preserve the system’s resilience following a failure. Therefore, research on controllers to support recovery and robustness after failure should be carried out as a follow-up to this study.

## Data Availability

Data available upon request to Insu Kim.

## Abbreviations

HVDC: High–voltage direct current
CB: Circuit breaker
DG: Distributed generator
MTDC: Multi–terminal direct current
SLG: Single line–to–ground
SSCB: Solid–state circuit breakers
LVDC: Low–voltage direct current
MVDC: Medium–voltage direct current
IGBT: Insulated gate bipolar transistor
MCC: Motor control center
SiC: Silicon carbides
TRV: Transient recovery voltage
